# A quest for universal anti-SARS-CoV-2 T cell assay: systematic review, meta-analysis, and experimental validation

**DOI:** 10.1038/s41541-023-00794-9

**Published:** 2024-01-02

**Authors:** Akshay Binayke, Aymaan Zaheer, Siddhesh Vishwakarma, Savita Singh, Priyanka Sharma, Rucha Chandwaskar, Mudita Gosain, Sreevatsan Raghavan, Deepika Rathna Murugesan, Pallavi Kshetrapal, Ramachandran Thiruvengadam, Shinjini Bhatnagar, Anil Kumar Pandey, Pramod Kumar Garg, Amit Awasthi

**Affiliations:** 1 Immunology Core Laboratory, Translational Health Science and Technology Institute, Faridabad, India; 2 Centre for Immunobiology and Immunotherapy, Translational Health Science and Technology Institute, Faridabad, India; 3 Jawaharlal Nehru University, New Delhi, India; 4 Translational Health Science and Technology Institute, Faridabad, India; 5 Department of Microbiology, AMITY University Rajasthan, Jaipur, India; 6 Pondicherry Institute of Medical Sciences, Puducherry, India; 7 ESIC Medical College and Hospital, Faridabad, India; 8 All India Institute of Medical Sciences, New Delhi, India

**Keywords:** Cellular immunity, Viral infection, Viral infection

## Abstract

Measuring SARS-CoV-2-specific T cell responses is crucial to understanding an individual’s immunity to COVID-19. However, high inter- and intra-assay variability make it difficult to define T cells as a correlate of protection against COVID-19. To address this, we performed systematic review and meta-analysis of 495 datasets from 94 original articles evaluating SARS-CoV-2-specific T cell responses using three assays – Activation Induced Marker (AIM), Intracellular Cytokine Staining (ICS), and Enzyme-Linked Immunospot (ELISPOT), and defined each assay’s quantitative range. We validated these ranges using samples from 193 SARS-CoV-2-exposed individuals. Although IFNγ ELISPOT was the preferred assay, our experimental validation suggested that it under-represented the SARS-CoV-2-specific T cell repertoire. Our data indicate that a combination of AIM and ICS or FluoroSpot assay would better represent the frequency, polyfunctionality, and compartmentalization of the antigen-specific T cell responses. Taken together, our results contribute to defining the ranges of antigen-specific T cell assays and propose a choice of assay that can be employed to better understand the cellular immune response against viral diseases.

## Introduction

Since 2020, multiple COVID-19 vaccines have been developed and subsequently given Emergency Use Authorization (EUA) to contain the pandemic. Vaccine efficacy studies implicate the role of cellular immunity in preventing severe COVID-19^1^, despite the reduction in vaccine-induced antibody titers over time, and loss of neutralizing capacity against Variants of Concern (VoCs). Moreover, earlier studies indicated that the T cell response to SARS-CoV-1 remains preserved over more than 10 years^2,3^, further emphasizing the role of T cells in long-lasting immunity.

Anti-SARS-CoV-2 antibody responses can be quantified, in convalescent and vaccinated individuals, in terms of their virus-neutralizing capacity as per World Health Organization (WHO) guidelines^4^. However, quantifying SARS-CoV-2-specific T cell responses is challenging due to the variability in T cell assays caused by differences in methods of peripheral blood mononuclear cells (PBMCs) isolation, type of stimulant (whole protein or peptide pool, 15mer or 11mer), the concentration of stimulants and co-stimulants (anti-CD40/anti-CD28/anti-CD49d, etc.) used, the density of cells used in assays, type of cell culture media used, duration of stimulation, and markers used to measure T cell activation, which together contributes to the observed differences in antigen-specific T cell responses. Therefore, a standardized and quantifiable assay to measure T cell responses to SARS-CoV-2 is much needed that can be utilized to determine the protective efficacy of vaccines and prior infection^5,6^. Moreover, it would aid in the designing of T cell-based next-generation vaccines for COVID-19.

Rising evidence has now underlined the relationship between the SARS-CoV-2-specific T cell response and protection against COVID-19 infection^7–12^. However, due to the unavailability of universal T cell assays, clinicians and policymakers primarily rely on antibody readout alone to determine the duration and efficacy of the vaccine and infection-induced immunity. Antibody responses wane over time and may not be a good indicator of protection against the VoCs of SARS-CoV-2^1^; hence measuring antibody response may not be sufficient for policy makers to decide on the need for and determining the interval for booster doses in large populations. Therefore, it is important to identify assays and markers that can determine T cell response, as they serve as reliable indicators of vaccine efficacy.

We performed a systematic review and a meta-analysis of studies that used various assays for measuring the infection and/or vaccine-induced anti-SARS-CoV-2 T cell responses. Based on our analysis, we defined a range of the readouts for the assays measuring T cell responses to SARS-CoV-2. These ranges were further confirmed by performing SARS-CoV-2 specific T cell assays (Activation Induced Marker (AIM) assay, intracellular cytokine staining (ICS) assay, and enzyme-linked immunosorbent spot (ELISPOT) assay) on convalescent and vaccinated individuals. The T cell assay readouts from three assays (AIM, ICS, and ELISPOT) from our experimental validation fell into the ranges obtained from meta-analysis. Secondly, these assays were performed on PBMCs of the same individuals, which provided us an opportunity to identify a correlation between them. Based on our data, we identify these assays to be correlated with each other, which provides the basis to identify a universal T cell assay.

## Results

**Fig. 1:**
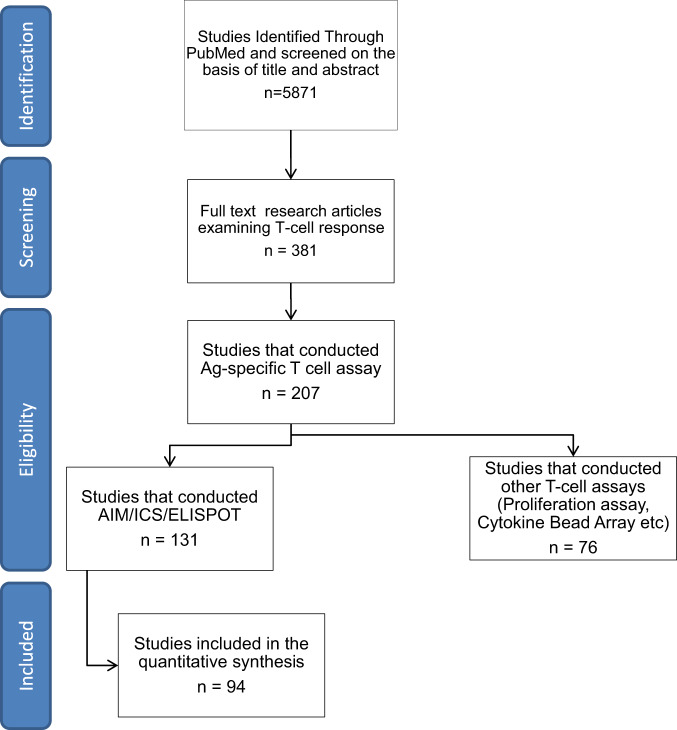
Process followed to shortlist studies for quantitative synthesis. Research articles were identified through PubMed search and screened on the basis of title and abstract. The identified articles were further screened by full-text examination to screen studies that reported antigen-specific T cell responses. The list was further narrowed down on the basis of eligibility according to the inclusion and exclusion criteria to finally include 91 research articles in the quantitative synthesis. All the research articles that performed AIM/ICS/ELISPOT Assay (n = 131) are summarized in Supplementary Data 5.

We performed the systematic review followed by a meta-analysis by screening research articles published between 01/01/2020 and 26/05/2022, as per PRISMA guidelines (Fig. 1). Our initial search resulted in 5871 publications that were further narrowed down by omitting overlapping studies obtained using different search keywords listed in Supplementary Table 1. We further screened these publications as per the inclusion and exclusion criteria listed in Table 1, and selected 94 studies for further in depth evaluation. We extracted datasets from these 94 studies, which measured the spike-specific T cell response by performing ELISPOT, AIM, and ICS for further analysis and evaluation, as given in Supplementary Data 1–4. Each of these three T cell assays were performed without a unified defined protocol prescribed by WHO or any other such regulatory body making it difficult to translate the readouts of these assays into T cell correlates of protection against COVID-19. The very first step was to define the readouts of these assays, which were performed in different laboratories across the globe without a single standardized validated protocol, and determine the ranges of the readouts of each of these assays. To do this, we performed a meta-analysis on the extracted datasets. Two independent assessors evaluated the risk of bias in the result synthesis, in any case of disagreement, the third assessor was consulted.

**Table 1:** Inclusion and Exclusion Criteria for Study Selection.

Assay	Inclusion Criteria	Exclusion Criteria
	Assay reported from January 1st, 2020, till May 26th, 2022, examining the human T cell response against the SARS-CoV-2 ancestral spike protein.	Studies analyzing pre-pandemic samples, non-spike-specific response, studies examining subsets besides CD4+ and CD8 + T cells; studies in animal models; assays where expanded T cells were used. Non-peer reviewed pre-prints
Additional assay-specific inclusion and exclusion criteria		
AIM	AIM assay performed between January 1st, 2020, till May 26th^,^ 2022, examining the surface expression of activation markers CD137 and OX40 on CD4+, and CD137 and CD69 on CD8 + T cells, upon stimulation with the SARS-CoV-2 ancestral (Wuhan isolate) spike protein.	Pre-pandemic (unexposed/unvaccinated), activation markers in subsets besides CD3 + CD4+ or CD3 + CD8+, surface markers besides CD137 and OX40 on CD4+ cells and CD137 and CD69 on CD8+ cells, surface markers for activation + intracellular cytokines, non-spike stimulation, single peptide stimulation, antigen-induced expansion of PBMC or long-term cultures (>48 h)
ELISPOT	Interferon gamma (IFNγ) ELISPOT assay reported between January 1st, 2020, till May 26th, 2022, examining the human T cell response against the SARS-CoV-2 ancestral spike protein.	Datasets reporting IFNγ ELISPOT response in PBMC samples of pre-pandemic unexposed/ unvaccinated individuals, sample size of fewer than three donors, or studies exclusively examining elderly populations. Datasets reported for PBMCs stimulated by a single peptide, non-spike peptide pools, whole protein ELISPOT data from studies that examined the T cell response <1 month after vaccination. S2 peptide pools were used for T cell stimulation.
ICS	Published data from Phase I/II/III/IV clinical trials, and efficacy examining the immunogenicity of any of the WHO EUL (Emergency Use Listing) vaccines until 25/05/2022 by ICS.	Datasets from non-WHO EUL vaccine efficacy study. Studies that do not evaluate vaccine efficacy.

### Defining the readouts of spike-specific AIM assays

Activation Induced Markers (AIM) are the surface proteins which are expressed on activated T cells post TCR stimulation. AIM assay identifies the magnitude of antigen-specific T cell response by measuring the frequency of T cells that express activation markers upon engagement of TCR with a specific peptide. Identification of antigen-specific CD4+ and CD8 + T cells can be determined using distinct sets of markers alone or in combination Supplementary Fig. 1. The most commonly used sets of markers for CD4 + T cells were OX40 and CD137, and CD69 and CD137 for CD8 + T cells in case of COVID-19 studies (Supplementary Data 1, 4, 5**)**. Therefore, we shortlisted 18 (12 studies^13–24^ for COVID-19 convalescent subjects and 8 studies^1,14,20–22,25,26^ for vaccinated subjects) research articles containing 89 different datasets (Supplementary Data 1) in which OX40 and CD137, CD69 and CD137 were used to measure spike-specific CD4+ and CD8 + T cells response respectively.

**Fig. 2:**
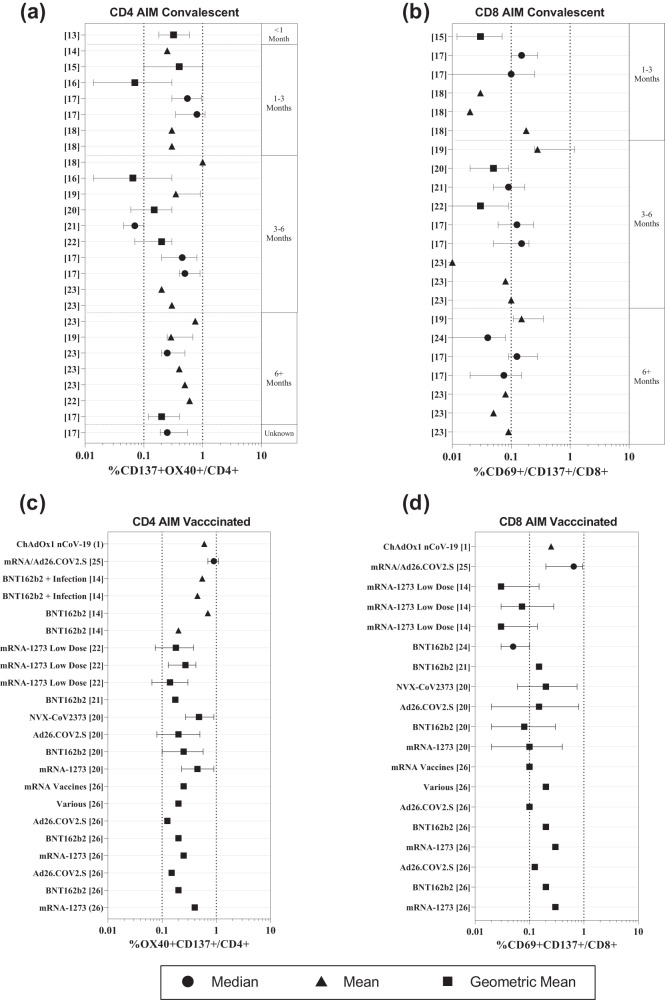
Forest plots of the Activation Induced Marker (AIM) T cell response in COVID-19 convalescent and vaccinated individuals. Forest plots depicting the range of readouts from AIM assays performed on (**a**, **b**) Convalescent cohorts and (**c**, **d**) vaccinated cohorts. Datapoints containing circles depict median; squares depict geometric mean; triangles represent mean. The y-axis represents the reference number of the individual datapoint represented. The x-axis represents the %frequency of AIM+ cells/frequency of parent (total CD4+ or CD8+ cells). For the full list of descriptive statistics for each study, please refer to Supplementary Data 1.

Of these 18 research articles, we shortlisted 12 studies^13–24^ with 48 datasets that examined samples from COVID-19 convalescent subjects. The central tendency of datasets from convalescent COVID-19 AIM studies were most commonly represented by mean (23/48), followed by median (15/48), and then geometric mean (GM) (10/48). We observed that the central tendencies of the frequencies of the AIM + CD4 T cells were significantly higher than the AIM + CD8 T cells (Supplementary Fig. 2). Based on our analysis, the range for each of these central tendencies was calculated. The range of central tendencies for CD4 + AIM was 0.02%–1% (mean), 0.07%–0.8% (median) and 0.065%–0.4% (GM), while the central tendencies for CD8 + AIM were 0.01%–0.25% (mean), 0.09%–0.15% (median), and 0.03%–0.05% (GM), (Fig. 2a, b**;** Table 2, Supplementary Data 1).

**Table 2:** Summary of the meta-analysis of the %frequencies of spike-specific T cell response determined by the activation induced marker assay.

		Mean		Median		GM	
		CD4	CD8	CD4	CD8	CD4	CD8
AIM Convalescent	No. of studies	3	2	2	2	6	3
	No. of datasets	12/26	11/22	7/26	8/22	7/26	3/22
	Range	0.02%–1%	0.01%–0.25%	0.07%–0.8%	0.09%/0.15%	0.065%/0.4%	0.03%–0.05%
AIM Vaccinated^a^	No. of studies	2	1	-	1	5	5
	No. of datasets	5/22	1/19	-	1/19	16/22	16/19
	Range	0.2%–0.9%	0.25%	-	0.05%	0.125%–0.48%	0.03%–0.3%

The number of studies and datasets that examined CD4+ and CD8 + T cells by AIM assays in vaccinated and convalescent cohorts using different measures of central tendencies, as depicted in Fig. 2a–d, and the range of the central tendencies for each group of datasets. The number of studies row represents the number of studies examining vaccinated or convalescent individuals used mean, median, or GM as their measure of central tendency. The number of datasets row represents the number of datasets represented by each central tendency, out of the total number of datasets extracted from various studies for each cohort as represented in Fig. 2a–d. The range row represents the range of each group of datasets that depicted %positive cells by mean, median, or GM.

^a^In studies examining AIM responses in vaccinated subjects, one study did not define the measurement of central tendency.

We further selected eight studies^1,14,20–22,25,26^ from 18 research articles that evaluated T cell response post vaccination. Forty-one datasets from 8 publications were analyzed for the range of central tendencies of CD4+ and CD8 + AIM. Geometric mean was the most common measure of midpoints (32/41), followed by mean (6/41) and then median (1/41), with two datasets from one study not mentioning their method of measuring central tendencies. Importantly, all these studies used whole spike peptide pools for T cell stimulation. We calculated the range of central tendencies for AIM assays in vaccinated individuals which were found to be 0.125%–0.48% (GM), and 0.2%–0.9% (mean) for CD4+ cells, and 0.03%-0.3% (GM) for CD8+ cells (Fig. 2c, d, Table 2, Supplementary Data 1). The range of readouts from AIM assays reported in studies using alternative markers to identify spike-specific T cells are summarized in Supplementary Fig. 3**(**Supplementary Data 5**)**.

### Defining the readouts of spike-specific ELISPOT assays

The IFNγ ELISPOT is one of the most widely used assays for evaluating the SARS-CoV-2-specific T cell response. Spike protein of SARS-CoV-2 was one of the most common antigens used in this assay to evaluate the anti-SARS-CoV-2 immunity. ELISPOT enumerates individual T cells that secrete IFNγ upon stimulation that can be measured as spot-forming units (SFU) per million PBMCs^27^. We shortlisted 62 studies for the meta-analysis to define the readouts of ELISPOT. Majority of the studies, 50 out of 62, containing 206 datasets, reported central tendencies as median in the form of SFUs/ million cells, hence we calculated the range of ELISPOT datasets that reported their mid-points as median.

**Fig. 3:**
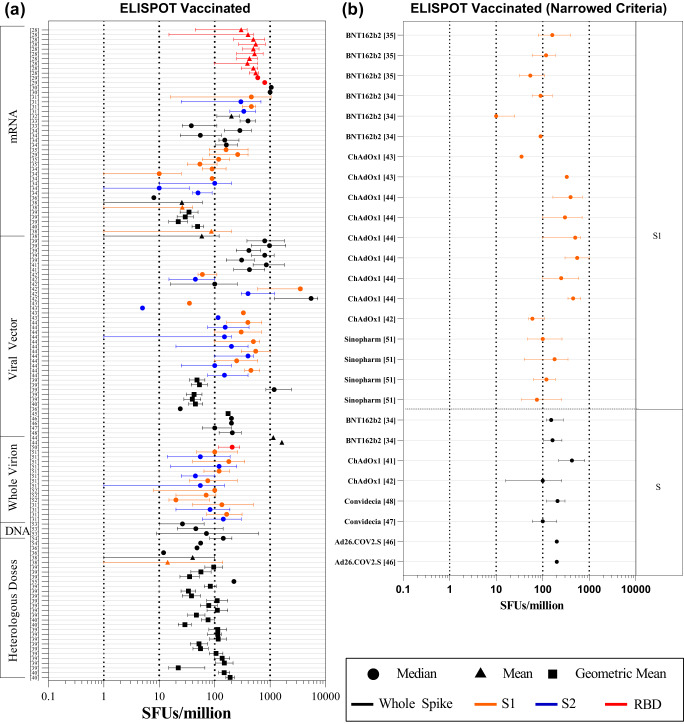

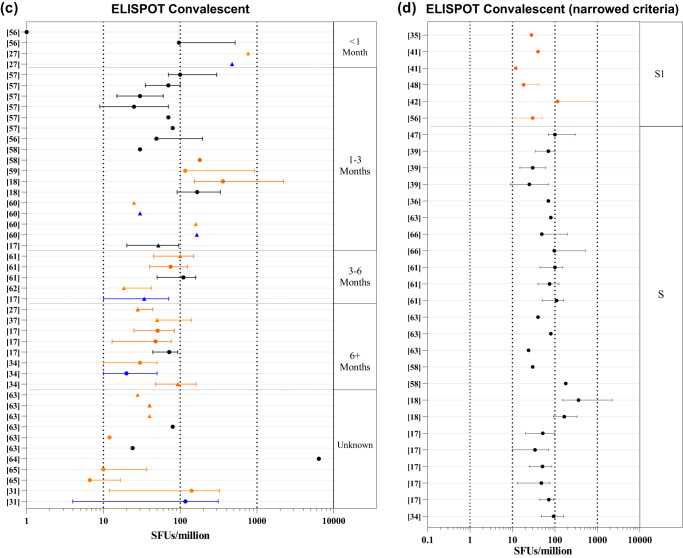
Forest plots of the IFNγ ELISPOT T cell response following COVID-19 vaccination or SARS-CoV-2 infection. Forest plots depicting the range of readouts from ELISPOT assays performed on (**a**) vaccinated cohorts, (**b**) vaccinated cohorts with narrowed criteria, (**c**) convalescent cohorts, and (**d**) convalescent cohorts with narrowed criteria. Datapoints containing circles depict median; squares depict geometric mean; triangles represent mean. Colors indicate the subunit(s) of the spike protein used for stimulation in each assay. Black represents the whole spike; orange represents S1 sub-unit; blue represents the S2 sub-unit; red represents the RBD. The y-axis represents the reference number of the individual datapoint represented. The x-axis represents the frequency of IFNγ spot forming cells (SFUs)/million PBMCs. For a full list of descriptive statistics for each study, see Supplementary Data 2.

For COVID-19 vaccinated individuals, the median range of 136 datasets from 28 research articles^28–55^ were found to be 5-1200 SFUs/million (Fig. 3a**;** Supplementary Data 2). We selected ChAdOx1 nCoV-19 (7 studies) and the BNT162b2 vaccines (9 studies) in which ELISPOT assay was used most frequently to determine the T cell responses. The range for IFNγ ELISPOT was found to be 8–460 SFUs/million and 5–1187 for the BNT162b2 and ChAdOx1 nCov-19 vaccines, respectively (Fig. 3a). Seventy out of 136 datasets used whole spike peptide pools followed by S1 peptide pools in 32/136 datasets (Supplementary Data 2). Thirty-one studies had concurrently evaluated T cell response against S1 and S2 domains of the spike protein. Comparison of these paired-datasets suggest a higher T cell response elicited against the S1 domain compared to the S2 domain (Supplementary Fig. 4). In addition, we found a broad range of responses in studies using ELISPOT assays to examine T cell response in subjects with co-morbidities or active COVID-19 (summarized in Supplementary Data 2).

In order to bring homogeneity in the readouts of ELISPOT datasets, we only focused to examine studies on COVID-19 vaccines that had received WHO EUL (Emergency Use Listing), and had demonstrated their effectiveness in real-world settings. Based on these criteria, we obtained 19 datasets from 6 research articles^34,35,42–44,51^ in which S1 peptide pool was used for stimulation and 8 datasets from 6 research articles^34,41,42,46–48^ used whole spike peptide pool for stimulation. The range of readouts of ELISPOT for the studies using S1 antigen was found to be 35–550, 10–119, 75–180 SFUs/million for ChAdOx1 nCoV-19, BNT162b2, and Sinopharm, respectively. Cumulatively, the range of IFNγ ELISPOT responses among WHO EUL vaccinated individuals was found to be 10–550 SFUs/million with IQR of 90–300 **(**Fig. 3b**)**.

Additionally, while calculating the range of readouts of ELISPOT in convalescent subjects^17,18,27,31,34,37,56–65^, we found a wider range in the antigen-specific T cell responses ranging from 1 to 361.3 SFUs/million cells. To further calculate the range of readouts of ELISPOT, we excluded one study^27^ in which spike-specific cells were expanded for 21 days before the enumeration of IFNγ spots, hence it resulted in median 6400 SFU/million (Fig. 3c). While most studies depicted central values of readouts in their datasets by median (50/62), other studies expressed them in mean or GM. To maintain uniformity in our analysis, we only considered the datasets whose central tendencies were expressed in median to calculate the above-mentioned range of ELISPOT readouts.

Similar to vaccine studies, we further focused on studies that used S1 or whole spike peptide pools for stimulation of T cells post 1 month of SARS-CoV-2 infection. In this analysis, we excluded studies which exclusively include elderly cohorts, as it is not clear whether elderly individuals would be able induce similar T cell response as younger adults. We obtained six datasets from five studies^35,41,42,48,56^ that used S1 peptide pool stimulation, and 24 datasets from ten studies^17,18,34,36,39,47,58,61,63,66^ that used the whole spike peptide pools for calculating range of readouts for ELISPOT in COVID-19 convalescent individuals. We found that the median range of ELISPOT readouts were 24–361.3 and 12–116 SFUs/million using whole spike stimulation and S1 stimulation, respectively (Fig. 3d). In summary, the range of IFNγ ELISPOT responses among convalescent individuals upon spike (both whole spike and S1 peptide pools) stimulation was 12–361 SFUs/million with IQR: 30–97.

### Defining the readouts of spike-specific ICS assays

**Fig. 4:**
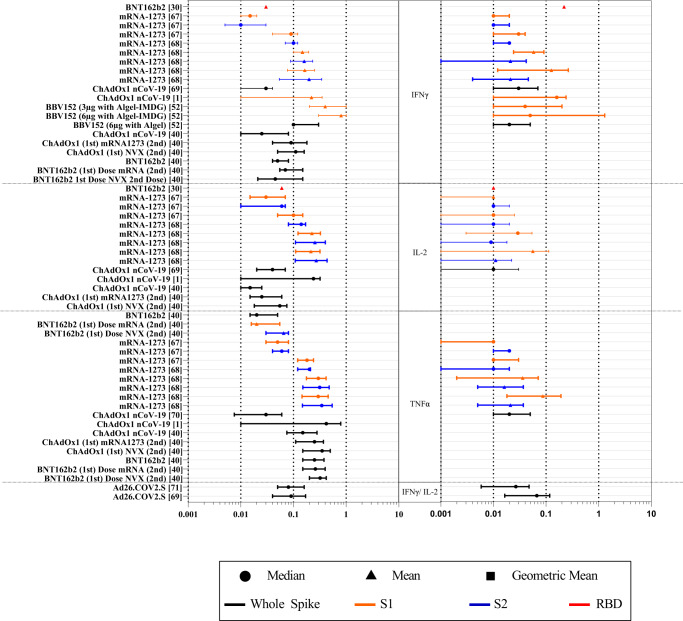
Forest plot of the Intracellular Cytokine Staining (ICS) T cell response in COVID-19 vaccinated individuals. Forest plot depicting the range of readouts from ICS assays performed in WHO EUL SARS-CoV-2 vaccine trial and efficacy studies. The left panel depicts the central tendencies of assays measuring CD4 + T cell intracellular cytokine production, whereas the right panel depicts CD8 + T cell intracellular cytokine production. The y-axis represents the reference number and vaccine type of the individual datapoint represented. The x-axis represents the %frequency of cytokine+ cells/frequency of parent (total CD4+ or CD8+ cells). Datapoints containing circles depict median; squares depict geometric mean; triangles represent mean. Colors indicate the subunit(s) of the spike protein used for stimulation in each assay. Black represents the whole spike; orange represents S1; blue represents S2; red represents the RBD. For a full list of descriptive statistics for each dataset, see Supplementary Data 3. The ranges depicted in the forest plot are further summarized in Supplementary Table 2.

ICS assays depict the level of intracellular cytokine expression in a population of cells. Although ICS is one of the most comprehensive approaches to identifying functional responses in antigen-specific T cells, it is prone to inter-laboratory variability due to technical challenges involved in performing this assay. Nonetheless, we calculated the range of readouts for ICS using data of clinical trials and efficacy studies of WHO EUL vaccines from which quantifiable ICS data could be extracted. Six COVID-19 vaccines from 9 studies^1,30,40,52,67–71^ were used to extract 96 ICS datasets. Eight studies examined IFNγ in both CD4+ and CD8 + T cells while one study examined IFNγ only in CD4 + T cells. Although all 9 studies reported antigen-specific IFNγ-producing cells, seven reported IL-2+ cells, and six reported TNFα expression (Supplementary Data 3). The central tendencies of datasets from the shortlisted studies were most commonly represented by median (58/96), followed by mean (38/96). The range of central tendencies for CD4 + IFNγ+ was found to be 0.01%–0.1% (median) and 0.03%–0.8% (mean) (Fig. 4a, Supplementary Data 3) while the central tendencies for CD8 + IFNγ+ were 0.016%–0.1% (median) and 0.02%–0.22% (mean) (Fig. 4b, Supplementary Table 2, Supplementary Data 3).

### Experimental interchangeability between AIM, ELISPOT, and ICS assays

**Fig. 5:**
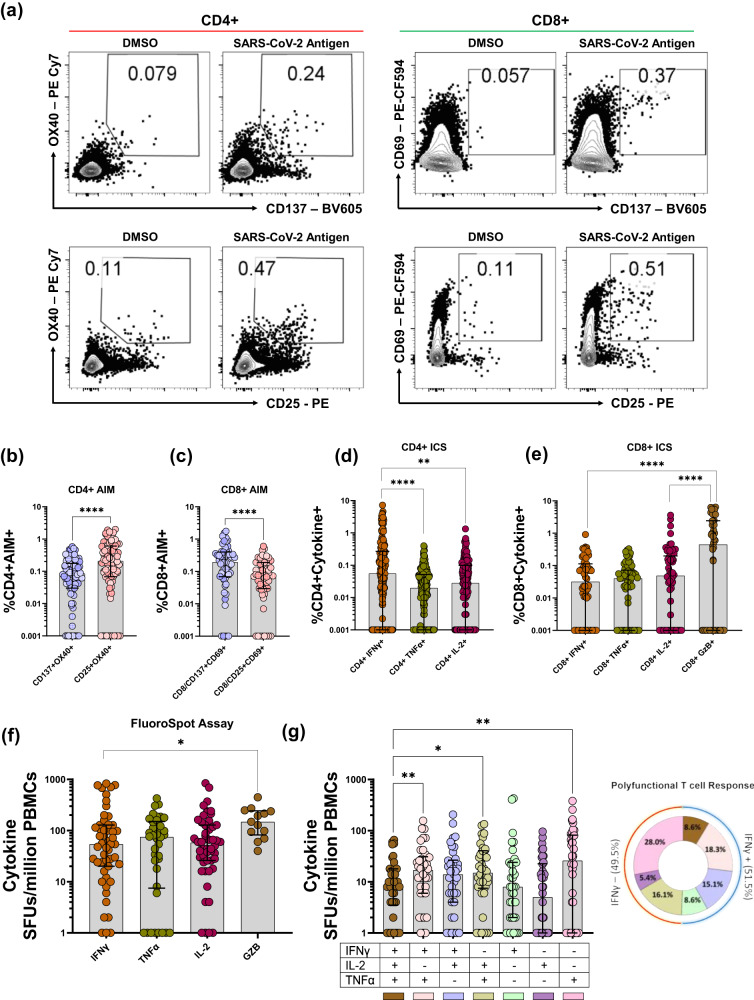
Experimental comparison of different antigen-specific T cell assays. **a** Representative FACS plots for AIM markers evaluated in CD4+ and CD8 + T cells. Comparison of T-cell responses in the validation cohort evaluated using different AIM and ICS markers: (**b**) CD4 AIM markers (n = 84) (**c**) CD8 AIM Markers (n = 58) (**d**) Cytokines evaluated by CD4 ICS (IFNγ: *n* = 188; TNFα: *n* = 188; IL-2: *n* = 162) and (**e**) Cytokines evaluated by CD8 ICS (n = 58 for each cytokine) (**f**) Cytokine spot forming units per million PBMCs evaluated by 3 color FluoroSpot Assays IFNγ/TNFα/IL-2 (n = 38) and IFNγ/IL-2/Granzyme B (n = 13) (**g**) Polyfunctional antigen-specific cytokine spot forming units per million PBMCs evaluated by three color FluoroSpot Assays IFNγ/TNFα/IL-2 (n = 38) are represented in all possible combinations of co-expression of cytokines. Each circle represents results obtained from one individual PBMC sample. Two-sided Wilcoxon-Signed Rank T test is performed for paired analysis. Friedman’s Test with Dunn’s multiple comparisons test is performed for non-parametric paired multiple comparison. Each data point shown was background subtracted. Bars and lines depict the median and IQR, respectively. GzB, Granzyme B; *p < 0.05; **p < 0.01, ***p < 0.001, ****p < 0.0001.

The above analysis in calculating the range of readouts for AIM, ELISPOT, and ICS in response to the spike antigen indicated high intra-assay variability resulting in wide range of readouts further complicating the task of defining a quantitative readout of T cell response as a correlate of protection. Although we made an attempt to reduce the intra-assay variability and identify studies that were performed with similar methodology and statistical analysis (Supplementary Fig. 5), no meaningful result could be drawn. Nonetheless, most studies performed these three different assays in order to understand the magnitude of cellular response in convalescent and vaccinated individuals. Since the readouts of these three primary T cell assays are driven by TCR stimulation, there is a possibility to define whether one of these three assays could be used as a universal T cell assay. To do this, we first tested whether AIM, ELISPOT/FluoroSpot, and ICS could be used interchangeably to measure the degree of T cell response either through measuring the intra or extracellular expression of IFNγ, TNFα, IL-2, and Granzyme B or surface expression of activation-induced markers. We performed the three assays on T cells from the same individuals and correlated their readouts to calculate the possibility of their interchangeability. PBMCs from individuals with vaccination and hybrid immunity (infection plus vaccinated) (Supplementary Table 3) were stimulated with the ancestral whole spike peptide pools or a peptide pool of immunodominant CD4+ and CD8 + T cell epitopes of SARS-CoV-2, and the antigen-specific T cell responses were measured concurrently by conducting AIM, ICS, and IFNγ ELISPOT or IFNγ/IL-2 /TNFα/Granzyme B FluoroSpot (Supplementary Figs. 6, 7). Our results on each of these three assays, i.e., AIM, IFNγ ELISPOT/FluoroSpot, and ICS (Fig. 5b–f) fell into the ranges we calculated from the meta-analysis (Fig. 2a–d; Fig. 3a, b; Fig. 4a, b).

ICS and IFNγ ELISPOT and FluoroSpot were conducted using defined parameters (cytokine staining for ICS, IFNγ secretion for ELISPOT, IFNγ, IL-2, and TNFα or IFNγ, IL-2 and Granzyme B for FluoroSpot). However, multiple surface markers were used to determine and report the frequency of CD4+ and CD8 + AIM (Supplementary Fig. 1b, c). Using the PBMCs from the validation cohort of 26 individuals, we first addressed the variability in AIM assays due to the combination of different activation markers. We tested the co-expression of the most commonly reported activation markers OX40, CD137, CD25, CD38, HLA-DR, and CD69 on CD4+ and CD8 + T cells upon spike activation in a subset of 26 samples (Supplementary Fig. 8). Our correlation data indicate that the activation marker CD38 and HLA-DR on both CD4+ and CD8 + T cells displayed poor correlation with a majority of T cell assays such as IFNγ ELISPOT, CD4/CD8 AIM, and CD4/CD8 IFNγ ICS. Therefore, CD38 and HLA-DR may not represent the actual population of antigen-specific T cells in this case and were excluded from further analysis.

**Fig. 6:**
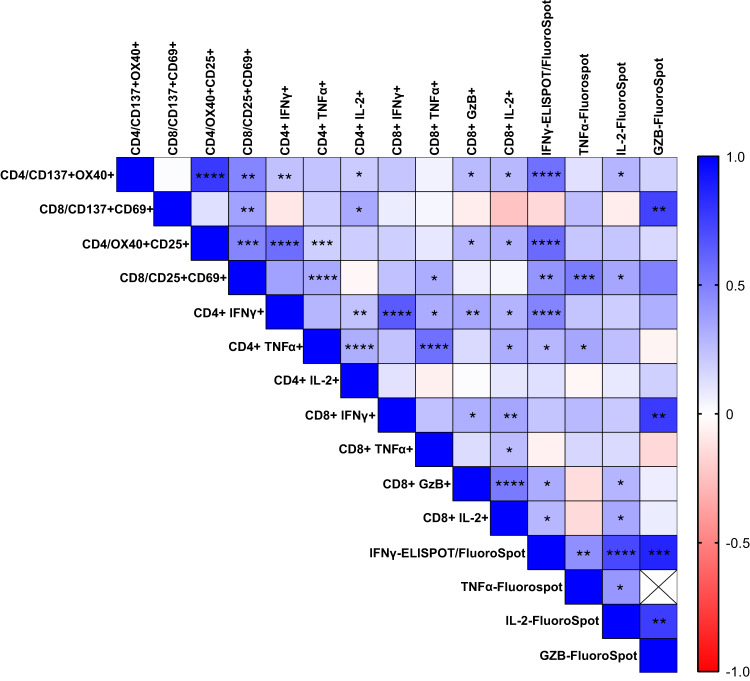
Experimental correlation of different antigen-specific T cell assays. Heatmap depicting the level of correlation between results from different T cell assays performed on PBMCs isolated from the same subjects (n = 193), based upon Spearman’s rank-order correlation. Stars represent *p*-values, and the intensity of color represents R-values (Spearman’s rank correlation coefficient); *p < 0.05; **p < 0.01, ***p < 0.001, ****p < 0.0001.

To further test the correlation among different CD4 + AIM and CD4 + ICS parameters, we tested the T cell responses in PBMCs of 109 vaccinated individuals upon stimulation with spike peptide pool (Supplementary Table 3). The spike-specific CD8 + T cell responses in our cohort of PBMC donors were sub-optimal. Therefore, to focus on both CD4+ and CD8 + T cell responses, we stimulated PBMCs (n = 58) with a peptide pool of immunodominant CD4+ and CD8 + T cell epitopes of SARS-CoV-2 to perform AIM, ICS, and three color FluoroSpot (IFNγ/IL-2/TNFα or IFNγ/IL-2/Granzyme B). The combined Spearman correlation analysis was performed for paired T cell responses evaluated in a total of 193 individuals (Fig. 6).

Correlation analysis showed that the frequencies of CD137 + OX40 + CD4+ cells correlated significantly with results from IFNγ ELISPOT/FluoroSpot (*p* = 1.45e-07; r = 0.55, *n* = 77). However, the correlation between CD137 + OX40 + CD4+ cells and CD4 + IFNγ + ICS was moderate (*p* = 0.0017; r = 0.24, *n* = 169). Therefore, we also used another activation marker, CD25, combined with OX40 to determine the frequency of antigen-specific CD4+ cells. The magnitude of CD25 and OX40 co-expression (median = 0.21%; IQR = 0.067–0.605), upon peptide stimulation, was significantly greater than that of CD137 and OX40 (median = 0.08%; IQR = 0.03-0.177) (*p* < 0.0001, *n* = 84) **(**Fig. 5b) Our experimental data suggest that CD137/OX40 or CD25/OX40 can be used to determine antigen-specific CD4 + T cells, as the correlation of these two CD4 + AIM markers was found to be significant (*p* = 5.5e-18; r = 0.77, *n* = 84). To further determine which of these two CD4 + AIM markers (CD137 + OX40+ and CD25 + OX40+) strongly correlated with other CD4+ and CD8+ assays, we performed correlation matrix analysis and found that the frequency of CD25 + OX40 + CD4 + AIM + T cells correlated with IFNγ ELISPOT assay (*p* = 3.03e-08; r = 0.58, *n* = 77). Moreover, the IFNγ ICS better correlated with the CD4 AIM assay when CD25 instead of CD137 was used (*p* = 1.4e-08; r = 0.57, *n* = 84). These data indicate that co-expression of CD25 and OX40, as compared to CD137 and OX40, may serve as better markers for measuring the SARS-CoV-2-specific helper T cell response in COVID-19 vaccinated or convalescent individuals.

Since CD25 is also expressed on Tregs, including CD25 co-expressing AIM markers may risk skewing the AIM response towards Tregs. To test this, we examined the expression of FOXP3 on CD25 + OX40 + CD4+ cells and compared them with CD137 + OX40 + CD4+ cells. We observed that in the PBMC samples we tested, the CD25 + OX40+ cells are predominantly FOXP3 negative (*p* < 0.01, *n* = 5, Supplementary Fig. 10) and are even higher than the proportion of FOXP3- cells in CD137 + OX40 + CD4+ cells. To further remove the FOXP3+ cells, the CD39+ population can be removed as described by the Kelleher group^72^.

Similar to the CD4 AIM assay, we used two sets of markers (CD137 + CD69+ and CD25 + CD69+) to determine the frequency of antigen-specific CD8 + T cells. Most studies, including those we reviewed in this paper, used CD137 + CD69+ surface markers (21/59) (Supplementary Fig. 1c, Supplementary Data 5). Based on our data on CD4 + AIM, CD25, in combination with OX40, showed a better response; therefore, we included CD25 with CD69 to determine the CD8 + AIM response. The median CD8 + AIM response evaluated by the combination of markers CD137 + CD69+ was significantly higher than CD25 + CD69+ (Fig. 5c) and correlated moderately (*p* = 0.005, r = 0.37, *n* = 58). To establish which of the above two CD8 + AIM markers (CD137 + CD69+ and CD25 + CD69+) better reflects the antigen-specific CD8 T cell population, we further correlated each of these CD8 + AIM markers with IFNγ ELISPOT, CD8 + ICS, CD4 AIM assay (both CD137 + OX40+ and CD25 + OX40+), Granzyme B ICS and Granzyme B FluoroSpot **(**Fig. 6**)**. Unlike CD137 + CD69+ (*p* = 0.29, r = -0.151, *n* = 51), the frequencies of CD25 + CD69 + CD8 + AIM correlated with IFNγ spots evaluated by the ELISPOT assay (*p* = 0.008; r = 0.366, *n* = 51, Fig. 6, Supplementary Data 6). Similarly, CD25 + CD69+, but not CD137 + CD69+, CD8 + AIM significantly correlated with the CD8 + T cell functional responses such as CD8 + TNFα+ intracellular expression (*p* = 0.014, r = 0.32, *n* = 58, Fig. 6, Supplementary Data 6). Since most of the SARS-CoV-2 specific T cell assays showed higher T cell reactivity in CD4+ than CD8 + T cells, it was imperative for us to test whether CD4 + AIM (both CD137 + OX40+ and CD25 + OX40+) correlates with CD8 + AIM (both CD137 + CD69+ and CD25 + CD69+). Our correlation matrix indicated that CD25 + CD69+, but not CD137 + CD69+, CD8 + AIM significantly correlated with both CD4 + AIM (CD137 + OX40+ (*p* = 0.007, r = 0.35, *n* = 58, Supplementary Data 6) and CD25 + OX40+ (*p* = 0.0009, r = 0.424, *n* = 58, Supplementary Data 6). These data indicate a good correlation between the TCR-based-activation induced marker response with the functional CD4 + T cell responses such as the IFNγ release.

For ICS assays, intracellular IFNγ, IL-2, TNFα and Granzyme B production was tested. The frequency of IFNγ + CD4 + T cells was significantly higher than CD4 + TNFα + (*p* < 0.0001, *n* = 188, Fig. 5d) and IL-2 + CD4 + T cells (*p* = 0.0019, *n* = 162, Fig. 5d), respectively. Interestingly, the median frequency of CD8+ Granzyme B+ cells upon SARS-CoV-2 antigen stimulation were fourteen-fold and nine-fold higher than the frequencies of CD8 + IFNγ + (*p* < 0.0001, *n* = 58) and CD8 + IL-2+ (*p* < 0.0001, *n* = 58), respectively (Fig. 5e). Similarly, the FluoroSpot assay shows that Granzyme B spot-forming cells were significantly higher than the IFNγ spot-forming units (*p* = 0.0134, *n* = 13, Fig. 5f). Although our meta-analysis showed that IFNγ ELISPOT was a preferred assay to enumerate the antigen-specific T cells, our experimental data indicates measuring IFNγ alone using ELISPOT/FluoroSpot covers about 51% of the antigen-specific T cells. Interestingly, about 49% of antigen-specific spot-forming units do not express IFNγ but express IL-2 and TNFα alone or in combination (Fig. 5g). This indicates that IFNγ ELISPOT significantly under-represent the SARS-CoV-2 specific T cell repertoire.

The intracellular IFNγ cytokine release from CD4 + T cells correlates significantly but moderately to the intracellular expression of TNFα (r = 0.284, *p* = 8.05e-05) and IL-2 (r = 0.236, *p* = 0.003). Similarly, the CD8 + IFNγ+ response correlates moderately with the CD8 + IL-2+ response (r = 0.348, *p* = 0.007) and CD8+GzB+ response (r = 0.311, *p* = 0.017) and does not correlate with CD8 + TNFα+ response (Fig. 6, Supplementary Data 6). Therefore, our results suggest that although IFNγ is a dominant cytokine in measuring functional T cell response against SARS-CoV-2, the addition of other cytokines, such as TNFα and IL-2 in combination with IFNγ, would represent an increased breadth of antigen-specific T cells repertoire with polyfunctionality.

Our study found that CD25 co-expressing AIM markers correlate better with cytokine responses than CD137 co-expressing CD4+ and CD8 + AIM markers. To determine the specificity of AIM+ markers, we stimulated PBMCs and sorted them based on surface markers for CD4 and CD8. We found that a higher percentage of antigen-specific T cells were identified using CD25 co-expressing activation markers compared to CD137 co-expressing activation markers (Supplementary Figs. 11, 12).

Despite several instances of correlation between multiple parameters of the different T cell assays, no single activation marker combination/cytokine could potentially be used as a surrogate to perform a comprehensive analysis of antigen-specific T cell response. Our results suggest that evaluating IFNγ release alone may not truly represent the status of antigen-specific T response in a given sample. We also show that the surface protein CD25 better correlates with other T cell assays and has higher specificity but lower sensitivity due to a relatively higher background.

## Discussion

In the absence of standard WHO-prescribed T cell assays, various regulatory bodies have primarily relied on measuring the antibody response in COVID-19-related policy decisions. However, recently it was reported that the combined magnitude of SARS-CoV-2-specific IFN-γ + T cell responses directed towards the SARS-CoV-2 structural proteins is a better correlate of protection against COVID-19 infection than antibody responses^8^. Understanding what range a T cell response falls into at a given time-point post-infection or vaccination could be an essential factor for policymaking in determining the need for booster doses, especially in cases where antibody titers are sub-optimal. Our meta-analysis of 94 research articles published between 01/01/2020 and 26/05/2022 shows that there is currently a wide disparity in the protocols used and the markers examined in three of these critical T cell assays (AIM, ICS, IFNγ ELISPOT), which makes it difficult to systematically define a specific range for T cell responses. A concerted effort to first define the protocol for a universal T cell assay, then examine long-term cellular immunity in a large cohort of healthy volunteers, should be made to precisely define the protective levels of T cell responses so that it can be used in the future for next-generation vaccine trials.

ELISPOT assays have been used extensively throughout the pandemic, with most studies using ELISPOT examining IFNγ secretion in response to spike antigens. Even within these assays, there was a large amount of variability in the methods used, including the time of cell stimulation, the concentration of stimulant used, the use of co-stimulants, and the number of cells seeded. The differences in protocol likely contributed to the significant differences in the range observed between similar types of studies examining similar cohorts of volunteers, making a direct comparison of ELISPOT assays between similar studies difficult. Although we have normalized the variations in the number of spots described to an extent by converting each dataset into spots/million PBMCs, the counting parameters and methods are rarely, if any, described by any study, further adding to the complexity in defining the ranges of the readouts of ELISPOT assays. Nevertheless, after narrowing the number of studies as described in the results (Fig. 3b, d) we were able to draw an inter-quartile range of ELISPOT responses to suggest that the T cell response for a healthy convalescent individual usually falls between 30–97 SFU/million and 90-300 SFU/million for vaccinated individuals, which may be an indication for a protective T cell response.

The IFNγ ELISPOT assay is known for its convenience, cost-effectiveness, and robustness. While the IFNγ ELISPOT assay is relatively less complex, which may offer the possibility of replicating results across different laboratories and streamlining the process, it is important to note that minimizing inter-assay variability necessitates a comprehensive effort encompassing harmonization of reagents, techniques, stages, and dedicated training. While T-SPOT®.COVID and Discovery kits are available for clinical settings^59^, they may not be affordable for clinical trials in low- and middle-income countries (LMICs). Thus, a standardized and universally accepted protocol for ELISPOT/FluoroSpot assays would be more beneficial. Our analysis has shown that relying solely on IFN gamma detection by ELISPOT cannot provide a complete understanding of the polyfunctionality and breadth of antigen-specific T cells. To better cover polyfunctional T cells and the breadth of T cell repertoire, especially in clinical settings without flow cytometry expertize, we suggest using a combination of IFNγ, IL-2, TNFα, and Granzyme B. The non-specific background of TNFα and Granzyme B is high (Supplementary Fig. 13), therefore, while the inclusion of TNFα assessment may increase the functional assessment of the assay, it may affect the specificity and limit of detection of the assay. Although our experimental analysis showed that the IFN-γ ELISPOT significantly correlated with the CD4 and CD8 AIM and ICS responses, the inability of ELISPOT to distinguish between responses from different T cell subsets unless sorted before the assay is performed remains a big challenge. Moreover, the fact that in our validation experiments, IFNγ ELISPOT did not significantly correlate with the CD8 IFNγ ICS response further underlines the drawback of ELISPOT/FluoroSpots’s inability to distinguish between different subsets of T cells. Therefore, ELISPOT/FluoroSpot Assays must only be employed when flow cytometry-based assays such as AIM/ICS are unavailable due to technical difficulties or logistic challenges involved in large-scale clinical studies or in clinical settings where results cannot be delayed.

IFN gamma release assay (IGRA) test is an attractive approach to determine the antigen-specific T cell response in clinical settings as well as for home sample collection. The efficiency of T cell assays using whole blood has been previously described. Some of the commercially available IGRA tests include the QuantiFERON SARS-CoV-2 assay^73^, Elecsys® IGRA SARS-CoV-2 test^74^, EUROIMMUN SARS-CoV-2 IGRA^75^, Wantai SARS-CoV-2 IGRA^76^, and COVID-19 Immuno-T™ test (ImmunoServ)^77^. While these assays primarily assess the release of IFN-γ after the stimulation of whole blood with either whole protein or peptide pools, they have the potential to utilize the same stimulated samples to evaluate the release of IL-2, TNF-α as well as other relevant cytokines according to the research question. The use of whole blood for T cell immunogenicity testing is a more attractive approach from a policy maker and a regulators point of view. The blood samples can be directly collected in a pre-antigen-coated blood collection tubes such as the QuantiFERON tubes or the SARS-CoV-2 Spike Protein TruCulture Tubes, moreover the COVID-19 Immuno-T™ test employs the capillary blood-based systems enables home collection of the sample from a finger prick. Such ease of application makes cytokine release assays (CRA) an attractive approach for T cell immunogenicity assessment. However, correlation studies have shown the relatively reduced sensitivity of whole blood IGRA such as the QuantiFERON SARS-CoV-2 assay compared to the IFNγ ELISPOT assay^78–80^. Moreover, there are other cellular sources of IFNγ and TNFα which may affect the results. Furthermore, cellular immunogenicity assessed in whole blood through IGRA may be affected by factors such as low lymphocyte counts (in the context of volume-based assays as opposed to standardized cell input in ELISpot) or the presence of immunosuppressive medication within the blood sample^80^. Another whole blood assay to evaluate the antigen-specific T cell response was reported by Schwarz et al. ^81^, who proposed to evaluate the expression of the CXCL10 gene following the incubation of whole blood with viral peptides, serving as an indicator of antigen-specific T cell responses^81^. These assays have the potential to facilitate population-level monitoring of cellular immunity to SARS-CoV-2 but require further validation from other research groups.

Activation Induced Marker (AIM) assay is another attractive approach for evaluating T cell response in COVID-19 vaccinated and convalescent individuals. Despite considerable assay variability, our quantitative synthesis suggests that the antigen-specific AIM + CD4 + T cells’ frequency commonly falls between 0.1–1% for total CD4 + T cells. In contrast, the median frequency of the antigen-specific CD8 + T cells is comparatively lower and falls between 0.01% and 0.3% (Fig. 2a–d). This difference is particularly distinct in convalescent individuals, consistent with the observations made by studies comparing T cell immunity among vaccinated and convalescent individuals^24^.

AIM assays could potentially be used in clinical settings due to their rapid nature (20–24 h cultures are commonly used, and data can be presented in less than 2 days from blood draw). It also provides an easy way to examine the T cell response in subsets such as CD4 + , CD8+, cTfh, TH1, TH2, TH17, and the memory subsets^24^, etc. as compared to ELISPOT or Cytokine release assays where the CD4 and CD8 component of T cell response cannot be differentiated. AIM assays have limitations - requiring specialized training and maintenance for a flow cytometer, and not providing information on T cell effector cytokine functions. The complexity of the assay makes automation challenging, and it can be prone to poor inter-lab proficiency.

More research is needed to determine the most effective combination of markers for T cell activation. Although our results show that most COVID-19 AIM studies reported using CD137-co-expression AIM, our experimental analysis found that the CD137 co-expressing CD8 + AIM responses did not correlate well with the results from other assays. Instead, CD25 expression on CD4+ and CD8 + T cells significantly correlated with the T cell functional responses. We further found that the use of CD137 co-expressing activation markers may underestimate the total frequency of antigen-specific T cells, while CD25 co-expressing activation markers have a higher rate of non-specific backgrounds. The role of CD25 as an activation marker to determine the antigen-specific T cells has been studied extensively^82–85^. Based on our experiments and previously published literature, we conclude that the CD137 AIM likely underrepresent the antigen-specific T cell population. However, a more detailed study is required to confirm these findings, test an array of activation markers for their specificity, and provide a combination of markers that most precisely represent the TCR-activation-based antigen-specific cells. In our study, no single parameter alone could precisely determine the frequencies of antigen-specific T cells. Therefore, until better assays for identifying antigen-specific T cells are widely available, a combination of AIM and ICS is currently the most suitable approach to understanding the breadth and functionality of antigen-specific T cells.

ICS assays carry many of the same advantages as AIM assays, with the added benefit of highlighting cytokines and surface markers, especially when performed in conjunction with AIM. ICS assays offer a qualitative analysis of T cell responses to an antigen. However, as a commonly used assay to measure T cell immunity, ICS is most suited to a specialized lab rather than for clinical use due to the long staining and fixing periods, the high number of fluorophore tagged antibodies, the necessity of a flow cytometer and personnel with the expertise to perform the requisite experiments and operate the cytometer^6^. Based on our data, AIM combined with ICS is the most comprehensive assay to measure the percent frequency and functionality of antigen-specific T cells. In addition, this combination of assays is also an indicator of various subsets of CD4 and CD8 T cells. Therefore, we advocate AIM + ICS as the assay of choice for determining the antigen-specific T cell response.

In the present study, we highlight the various variabilities in the T cell assays employed by different labs and different studies from the same lab. One such variability is the time of stimulation which has ranged from 4–6 h in some ICS assays to 18–48 h in AIM, ICS, and ELISPOT Assays (Supplementary Data 2, 5). The optimal time for evaluating the response depends on multiple factors such as the type of antigen (for example, peptide pool stimulation requires lesser time compared to whole protein), type of cytokine being evaluated (for example, Granzyme B FLUOROSpot may require 48 h stimulation) or the type of activation marker being analyzed. The optimal surface expression for activation makers has been recently summarized by Poloni et al.^86^. For rare event flow cytometry assays such as the antigen-specific T cell AIM or ICS assay, sensitivity and specificity should be carefully evaluated in assay development and validation. It is important to note that increasing the flow rate can lead to a higher rate of coincidence in some systems, which can result in a decrease in assay sensitivity. Therefore, to achieve high sensitivity and specificity, it is crucial to carefully evaluate the impact of sample concentration, acquisition rate, and acquisition time^87,88^.

In our meta-analysis we also observed a substantial variation in the positivity threshold selected in the various published studies. The antigen-specific T cell response (AIM, ELISPOT, and ICS) is calculated by background-subtraction of the values obtained after antigen stimulation with the unstimulated autologous sample. Majority of the studies designate participants with positive values post-background subtraction as responders^1,29,89,90^. In the case of AIM assay, stimulation index^15,21^ has been used to differentiate responders vs non-responders. Nonetheless, the threshold for flow cytometric assays such as the AIM and ICS assays were arbitrarily calculated differently by different groups based on the difference in number of events observed^91^ or the percentage of events^20^ observed between stimulated and unstimulated samples.

During antigen-specific T cell stimulation, the type of stimulant also plays an important role. Peptides and peptide pools are the stimulants of choice for most investigators because of the rapid stimulation of both CD4 and CD8 T cells in vitro. CD8 + T cell responses reported in vitro often may not reflect the magnitude of the same response in vivo, as 15-mer peptides are commonly used to stimulate T cells, which are larger than the 9-11-mer peptides that bind MHC class I molecules. Additional processing steps must occur to present these peptides to CD8 + T cells, which may result in a dampened response^85^, therefore suggesting that 15mer peptides are not ideal for measuring the Ag-specific CTL response. Indeed, a drop of over 20% has been reported in the antigen-specific CD8 response using 15mer peptide pools^92^, which is consistent with about 17% drop observed in our AIM assay meta-analysis (Supplementary Fig. 2).

In our meta-analysis, we observed that the central tendencies were represented by different groups as mean, median, or geometric mean. Since all the antigen-specific T cell responses are calculated by subtracting the DMSO-stimulated wells from antigen-stimulated wells, the data is non-parametric and broadly ranged. Therefore, measurement of central tendencies must be performed using geometric mean or median, whereas arithmetic means must be avoided.

The pandemic has led to new therapeutics and vaccine platforms and improved our understanding of anti-viral human immunology, laying the groundwork for future breakthroughs. For studying the antigen-specific T cells, the use of fluorescently conjugated peptide class I and class II MHC multimers - tetramers^93^, pentamers^94^, and/or spheromers^95^ are the assay of choice for viral immunologists. However, MHC-multimer-based flow cytometry is limited to individuals with particular HLA haplotypes, making it unsuitable for evaluating T cell responses at the population level due to HLA polymorphism. TCR sequencing assays based on next-generation sequencing (NGS) in conjunction with machine learning algorithms is a rapidly advancing and potent field to identify a T cell response against an antigen^6,96^. However, these molecular assay workflows are currently in their preliminary stage. It is necessary to validate these methodologies in larger datasets from diverse racial groups and individuals with varying HLA types, as these factors can potentially affect the analysis.

This study analyzed results from studies that performed the three most commonly used assays during the pandemic. To maintain uniformity in our analysis, we did not examine AIM assays that investigate T cell immunity as a percentage of memory T cell subsets or cells co-expressing surface and intracellular markers. We also did not study other T cell assays in detail, such as proliferation analysis by CFSE staining, which is unsuitable as potential gold-standard T cell assays due to the extensive protocols they require compared to the three examined in this study.

The argument underlining the necessity of a standardized protocol for gauging T cell immunity has been previously made^6,97^. Most such arguments also focus on the practical and logistic advantages and drawbacks of the assays. Therefore, we also examined how these assays correlate to see if they are genuinely interchangeable from a scientific viewpoint.

While some reports suggest that ELISpot assays are highly specific, the AIM assay was reported to be more sensitive and had a higher signal-to-noise ratio^98^. Conversely, other studies have reported ELISpot to be more sensitive than the ICS assays^99^, particularly in the detection of memory T cells, which produce lower amounts of cytokine upon reactivation^100,101^. The presence of cross-reactive T cell responses to SARS-CoV-2 proteins, resulting from exposure to common-cold coronaviruses, has been reported extensively^2,13,102^. This phenomenon significantly complicates the differentiation between true positives and negatives, further exacerbating the challenge of comparing the overall sensitivity and specificity of these SARS-CoV-2-specific T cell assays. As such, the determination of the relative specificity and sensitivity of T cell assays is a crucial topic, one that underscores the need for a meticulously designed study that considers the intricacies and potential confounders inherent to each assay.

Our results showed that although AIM assays, ICS assays, and ELISPOT/FluoroSpot assays performed on the same individual’s PBMCs correlate with each other, a substantial proportion of antigen-specific T cells are underrepresented by evaluating a single parameter such as the IFNγ response. Therefore, in scenarios where flow cytometric assays are not feasible, multi-color ELISPOT/FluoroSpot assays may be employed. Otherwise, AIM, in combination with ICS assays, should be the assay of choice to determine the percent frequency and functionality of the antigen-specific T cells.

## Methods

### Systematic review and meta-analysis

The PubMed database was searched for research articles published between 01/01/2020 and 26/05/2022 using the keywords for each of the assays (AIM, ICS, ELISPOT) as listed in Supplementary Table 1, and further screened based on the inclusion and exclusion criteria listed in Table 1.

Data denoting mean or median SFUs (spot-forming-units)/10^6^ cells for ELISPOT was extracted as reported (mean/median SFUs/10^6^ cells). In studies that reported ELISPOT data in any format other than SFUs/10^6^ cells, it was extrapolated to make it SFU/10^6^ cells. Data for AIM and ICS were represented by mean, geometric mean, or median % positive cells based on the surface expression of AIM markers or cytokine-secreting CD4+/CD8 + T cells, respectively. Wherever exact numbers of each of these above-mentioned assays were not mentioned in the text/figure legend, data points were estimated from graphs wherever possible, as described previously^5^. The data was collected by two independent researchers (A.B. & A.Z.) and reverified (R.C., A.A.) in case of disagreement, consensus was reached on inclusion or exclusion by discussion and if necessary, the third researcher (A.A.) was consulted. The systematic review was not registered and the study protocol was not prepared.

The most commonly examined T cell subsets from the literature were CD3 + CD4+ and CD3 + CD8+, and their respective cytokine or activation marker expression have been analyzed in the present study. However, numerous studies also examined early activation of T cell memory subsets such as Tem and Temra, as well as surface markers in conjunction with intracellular markers. AIM assays depicting Ag-specific T cells as a percentage of total memory CD4+ or CD8+ subsets such as Tem were excluded from this analysis as they depict a different range of results from the majority of studies, which examined Ag-specific T cells by AIM assays as a percentage of the entire CD4+ or CD8+ populations. For similar reasons, studies using the Stimulation Index to depict AIM+ cells are excluded from Fig. 1 but included in Supplementary Data 5.

### Human ethics statement

All experiments were performed as per the suggested guidelines of the Institutional Ethics Committee (Human Research) of our institute (Ref No: THS 1.8.1 (97)). Blood was collected from COVID-19 recovered subjects and vaccinated individuals at least 1 month after recovery or vaccination, and written informed consent was taken. The participant characteristics are summarized in Supplementary Table 3.

### Peptide pools

15-mer peptide pools spanning the length of the SARS-CoV-2 spike protein (ancestral strain) with 11 amino acid overlaps (157 + 158 peptides, JPT, PepMix, Cat. No. PM-WCPV-S-1) (ancestral) were used at a concentration of 2 µg/ml to stimulate spike-specific T cells detected by ELISPOT, AIM, and ICS assays. To further study the CD8 responses, a subset of samples was stimulated with a pool of 103 peptides (defined HLA class I & II-restricted T-cell epitopes) from selected proteins of SARS-CoV-2 (JPT, PepMix™ Pan-SARS-CoV-2 Select, Cat. No. PM-Pan-SARS2select01-1).

### PBMC isolation

PBMCs were isolated using the Ficoll-Paque density gradient centrifugation method. A ratio of 1:1 of the blood sample diluted with PBS was layered on top of Lymphoprep™ and centrifuged at 800 g for 30 min without brakes at 25°C. The PBMC layer was collected by pipetting along with the remaining plasma and transferred to a 50 ml tube, which was filled up to 50 ml with complete media and centrifuged at 300 g at 4 °C for 15 min. The cells were resuspended in 5 ml of complete RPMI, counted using a hemocytometer and Trypan Blue staining, and the number of cells was recorded^1^. The PBMCs were aliquoted in freezing media (FBS + 10%DMSO) and cryopreserved in liquid nitrogen. The PBMC samples were thawed at 37 °C in pre-warmed complete RPMI media and were rested for at least 2 h at 37 °C before stimulation. The live PBMCs in each sample were counted and were simultaneously seeded for AIM/ICS and ELISPOT/FluoroSpot Assays. Except for the IFN-γ/IL-2/TNF-α FluoroSpot, complete RPMI (RPMI 1640, 10% heat-inactivated FBS, 1 % nonessential amino acids, 1 % sodium pyruvate, 2 mM L-glutamine, 5 × 10 − 5 M β-mercaptoethanol) was used for PBMC culture.

### ELISPOT assay

ELISPOT assays were performed as previously described^1,103^. Briefly, PBMCs were seeded in pre-coated ELISPOT plates at 0.25 million cells/well, as per the manufacturer’s instructions (Human IFN-γ ELISpot PLUS kit (ALP) (Product Code: 3420-4APT-10)). An equimolar concentration of dimethyl sulfoxide (DMSO) was used as a negative control. Anti-CD3 was used as a positive control. The SARS-CoV-2 Spike peptide pool (2 µg/ml) was added to the test wells to evaluate the spike-specific T cell response. After stimulation for approximately 20 h, the plates were processed, and spots were enumerated using an Immunospot® S6 Ultimate M2 machine. The number of spots forming units (SFUs) per million cells was calculated by multiplying the background subtracted spots per well by four. Samples with a low anti-CD3 response (<45 SFUs/million PBMCs) were excluded from the analysis.

### FLUOROSPOT assay

Ex-vivo SARS-CoV-2-specific IFN-γ/IL-2/TNF-α FluoroSpot (ImmunoSpot Human kit, Cleveland, USA) and IFN-γ/GranzymeB/IL-2 FluoroSpot (MabTech Human FluoroSpotPLUS kit, Sweden) assays were carried out to determine the levels of virus-specific cytokine-producing cells in a subset of PBMC samples (IFN-γ/IL-2/TNF-α: *n* = 38; IFN-γ/GranzymeB/IL-2: *n* = 13) following the manufacturer’s protocols. For the IFN-γ/IL-2/TNF-α FluoroSpot assay, capture antibody pre-coated wells were seeded with PBMCs at a concentration of 0.25 million/well and incubated for approximately 20 h at 37°C and 5% CO2 with appropriate stimulants/co-stimulants in CTL serum-free media supplemented with L-glutamine, 1% Penicillin: Streptomycin solution (Thermo Scientific). For the IFN-γ/GranzymeB/IL-2 FluoroSpot assay, pre-coated wells were conditioned with sterile complete RPMI medium for 30 min. They were seeded with PBMCs at a concentration of 0.25 million/well and incubated for approximately 45 h at 37 °C and 5% CO2 with appropriate stimulants/co-stimulants in complete RPMI 1640 media.

To test the SARS-CoV-2 specific response, the PBMCs were stimulated with the virus-specific pool of immunodominant HLA class I & II-restricted T-cell epitopes of SARS-CoV-2 proteome (PanSARS-CoV-2 PepMix peptide pool, JPT Peptide Technologies, Germany) at a concentration of 1 µg/ml of each peptide. As an internal negative control, each PBMC sample was also cultured with an equimolar concentration of DMSO, whereas for positive control, PBMCs were stimulated with 5 µg/ml PHA. Co-stimulants anti-human CD28, anti-human CD49d, and anti-human CD40 (UltraLeaf purified, BioLegend, USA) at a final concentration of 2 µg/ml were added in the test and negative control wells. Post incubation, the plate was developed as per the manufacturer’s guidelines, and the spots were quantified using an Immunospot® S6 Ultimate M2 analyzer (CTL, Cleveland, USA). The number of spots forming units (SFUs) per million cells was calculated by multiplying the background subtracted spots per well by four. Samples with a low PHA response (<45 SFUs/million PBMCs) were excluded from the analysis.

### AIM and ICS assays

AIM and ICS assays were performed as previously described^1,89^. Briefly, cryopreserved PBMCs were thawed, rested for at least 1 h, and subsequently cultured in U-bottom plates at 1 million cells/200 µl/well. Before adding peptides, the PBMCs were blocked at 37 °C for 15 min with 1 μg/ml of anti-CD40 mAb (Biolegend, USA). The cells were co-stimulated with 1 μg/ml of anti-CD28/CD49d cocktail (BD Biosciences, USA; Cat. No. 347690). The cells were stimulated with 2 μg/ml of peptide pool, and an equimolar concentration of DMSO was used as a negative control. As a positive control, cells were stimulated with 5 μg/ml PHA (Phytohemagglutinin L, Roche). After 20 h of culture, monensin (GolgiStop^TM^, BD Bioscience, USA) and anti-CD137 BV605 (Biolegend, USA, Cat. No. 309822, 1:100) were added for the last 4 h. The cells were stained with the following antibodies: CD4 FITC (561842, 1:100), CD8 Alexa Fluor® 700 (344724, 1:100), OX40 PECy7 (350012, 1:100), CD69 PE/Dazzle™594 (310942, 1:100), CD25 PE (353204, 1:200), CD38 BV711 (303528, 1:100), HLA-DR BV785 (307642, 1:50), TNF-α PerCP/Cyanine5.5 (502926, 1:100), and IFNγ APC (502512, 1:100). Live cells were determined using Zombie NIR™ Dye (1:500) (all antibodies and dyes purchased from Biolegend, USA). The acquisition of the samples was performed on a fluorescence-activated cell sorter (FACS) Symphony™ instrument (BD Biosciences), with BD FACSuite software version 1.0.6, and the analysis of the results was done using FlowJo software version VX (FlowJo LLC, BD Biosciences). The activation markers and cytokines frequencies were calculated by subtracting the respective frequencies of peptide-stimulated wells by DMSO-stimulated wells of the same sample.

### T cell expansion and restimulation assay

Freshly isolated PBMCs from healthy individuals (n = 4) with hybrid immunity against SARS-CoV-2 were expanded by stimulating with SARS-CoV-2 spike antigen, as described previously^104^ (Summarized in Supplementary Fig. 11a). Briefly, 0.1 million cells/200 µl/well PBMCs were cultured for the first 24 h in the presence of 1000 IU/mL GM-CSF (Miltenyi Biotech), 500 IU/mL IL-4 (Miltenyi Biotech), and 50 ng/mL Flt3-L (Miltenyi Biotech) at 37 °C. The next day, 100 µl of the culture supernatant was gently replaced and cells were stimulated with peptide pools (2 µg/ml of each peptide), 10 µM R848 (Sigma-Aldrich), 100 ng/ml LPS (Invivogen), and 10 ng/ml IL1β (Peprotech). After 24 h, 100 µl of the culture supernatant was gently replaced to culture the cells in the presence of 10 IU/mL IL-2, 10 ng/mL IL-7, and 10 ng/mL IL-15 (Miltenyi Biotech). The cells were expanded for 6 days where 100 µl/well of culture medium containing IL-2, IL-7, IL-15 and peptide pool was replaced every 48 h. After 6 days, the cytokine and peptide stimulation were removed, and cells were cultured in complete media for 72 h. Next, the cells were restimulated with a SARS-CoV-2 spike peptide pool in the presence of anti-CD49d/CD28 (BD Biosciences, USA) and cultured for 20 h. After incubation, the stimulated cells from each well were pooled and aseptically stained for viability – Zombie^TM^ NIR (Biolegend, USA), CD3-APC (Biolegend, USA, Cat. No. 317318), CD4-PacificBlue (Biolegend, USA, Cat. No. 317429), CD8-PERCP-Cy5.5 (BD Pharmingen™ USA, Cat. No. 565310), CD137-BV605 (Biolegend, USA, Cat. No. 309822), CD69-FITC (BD Pharmingen™ USA, Cat. No. 555530), and CD25-PE (Biolegend, USA, Cat. No. 353204) at 1:50 each, diluted in sterile FACS Buffer. The cells from each individual were equally divided into two parts and were two-way sorted into CD25+ co-expressing AIM + CD4+ (CD25 + OX40+) and CD8 + (CD25 + CD69+) cells or CD137+ co-expressing AIM + CD4 + (CD137 + OX40+) and CD8 + (CD137 + CD69+) cells. The FACS sorted cells were counted and seeded with Mitomycin C fixed autologous cells at 1:100 cell concentration in U-bottom 96 well plate in the presence of IL-2, IL-7, IL-15, and peptide pool. The cells were cultured and expanded such that IL-2, IL-7, and IL-15 containing media were added every 3–4 days. The peptide pool was added after every 7–8 days at 1 µg/ml concentration. After approximately 30 days, the cytokine and peptide stimulation were removed, and cells were cultured in complete media for the next 96 h. Next, the cells were restimulated with the SARS-CoV-2 spike peptide pool, anti-CD49d/CD28 co-stimulation, and cultured for 20 h. The stimulated cells were washed and analyzed by flow cytometry.

### Statistics

Data visualization and statistical analysis were performed using GraphPad Prism 9.0 and FlowJo XV. Correlation analysis was performed using Spearman’s Correlation Test. A two-sided Wilcoxon signed rank *t*-test was employed for paired non-parametric analysis. Unless otherwise stated, bar graphs depict the minimum and maximum values. *represents *p* ≤ 0.05; ** represents *p* ≤ 0.01; *** represents *p* ≤ 0.001; **** represents *p* ≤ 0.0001.

### Reporting summary

Further information on research design is available in the Nature Research Reporting Summary linked to this article.

### Supplementary information

Supplementary Information File

Supplementary Data 1

Supplementary Data 2

Supplementary Data 3

Supplementary Data 4

Supplementary Data 5

Supplementary Data 6

REPORTING SUMMARY

## Data Availability

All raw and processed data is presented in the supplementary tables. Any additional information that is required to reanalyze the data reported in this paper is available from the lead contact upon request.
