# Supplementary material for: A quest for universal anti-SARS-CoV-2 T cell assay: systematic review, meta-analysis, and experimental validation
Source: NPJ Vaccines. 2024 Jan 2;9:3. doi: 10.1038/s41541-023-00794-9 (PMC10762233; doi:10.1038/s41541-023-00794-9)
Supplement: Supplementary file 1 — Supplementary Information File [file 41541_2023_794_MOESM1_ESM.pdf]

SUPPLEMENTARY INFORMATION FILE

Title: A quest for universal anti-SARS-CoV-2 T cell Assay: systematic review, meta-analysis, and experimental validation.

Akshay Binayke[1,2,3]†, Aymaan Zaheer[1]†, Siddhesh Vishwakarma[1], Savita Singh[4], Priyanka Sharma[1], Rucha Chandwaskar[5], Mudita Gosain[4], Sreevatsan Raghavan[4], Deepika Rathna Murugesan[4], Pallavi Kshetrapal[4], Ramachandran Thiruvengadam[4,6], Shinjini Bhatnagar[4], Anil Kumar Pandey[7], Pramod Kumar Garg[4,8], Amit Awasthi[1,2*]

1 Immunology Core Laboratory, Translational Health Science and Technology Institute, Faridabad, India;

2 Centre for Immunobiology and Immunotherapy, Translational Health Science and Technology Institute, Faridabad, India;

3 Jawaharlal Nehru University, New Delhi, India

4 Translational Health Science and Technology Institute, Faridabad, India;

5 Department of Microbiology, AMITY University Rajasthan, Jaipur, India.

6 Pondicherry Institute of Medical Sciences, Puducherry, India;

7 ESIC Medical College and Hospital, Faridabad, India;

8 All India Institute of Medical Sciences, New Delhi, India

*Corresponding and Lead author. Email: aawasthi@thsti.res.in

† Equal Contribution

Supplementary Figures:

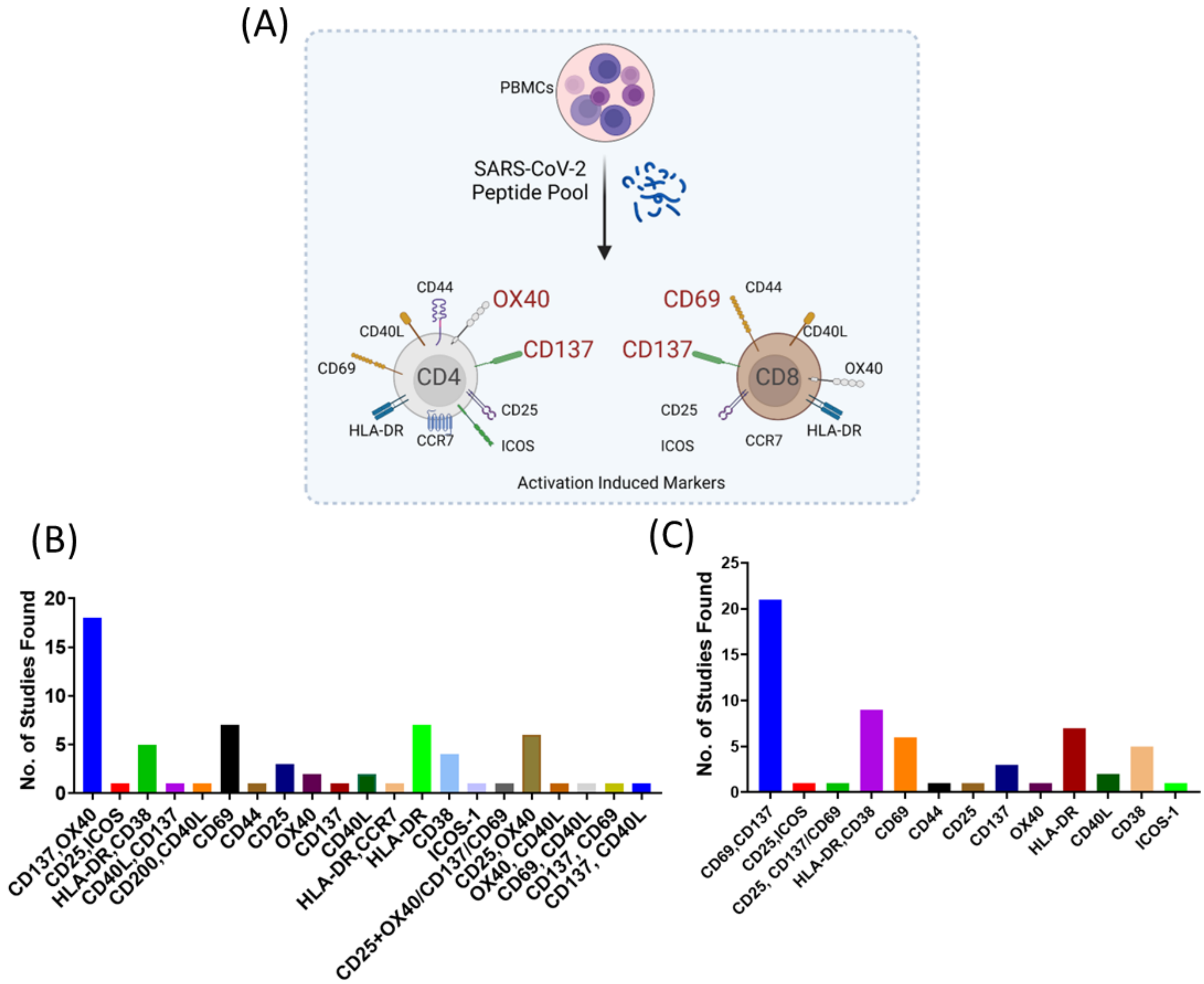

Supplementary Figure 1: Different Activation-induced markers (AIM) used to study the antigen-specific T cell response. (A) Schematic diagram of different activation markers reported in the literature[17,23,109-157] to study SARS-CoV-2 specific T cells upon peptide stimulation. Markers in red font represent the most commonly reported activation markers; created with BioRender.com. (B-C) Bar graph illustrating the different combinations of activation markers utilized by studies and the number of studies found in our literature review that reported the use of a combination of AIM markers for (B) CD4+ T cells and (C) CD8+ T cells. For a complete list of descriptive statistics for each study, see **Supplementary Table 6.**

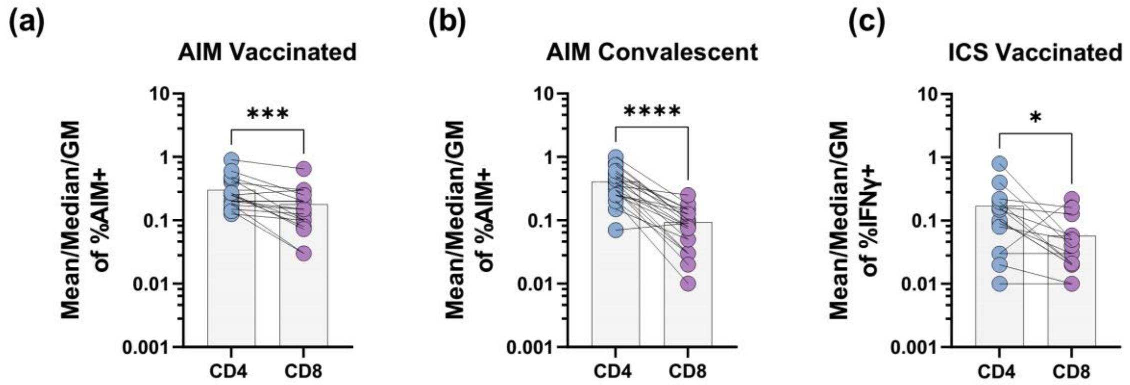

Supplementary Figure 2: Paired comparison between the antigen-specific CD4 and CD8 T cell response evaluated in the same study. Each dot represents a central tendency (mean/median/geometric mean(GM)) reported by the individual study. The AIM response is calculated by the % co-expression of OX40/CD137 for CD4 cells and CD69/CD137 for CD8 cells. Bars represent mean values of the dataset. Wilcoxon-signed rank two-sided paired t-test is performed for statistical analysis. * p<0.05; ** p<0.01, *** p <0.001, **** p<0.0001.

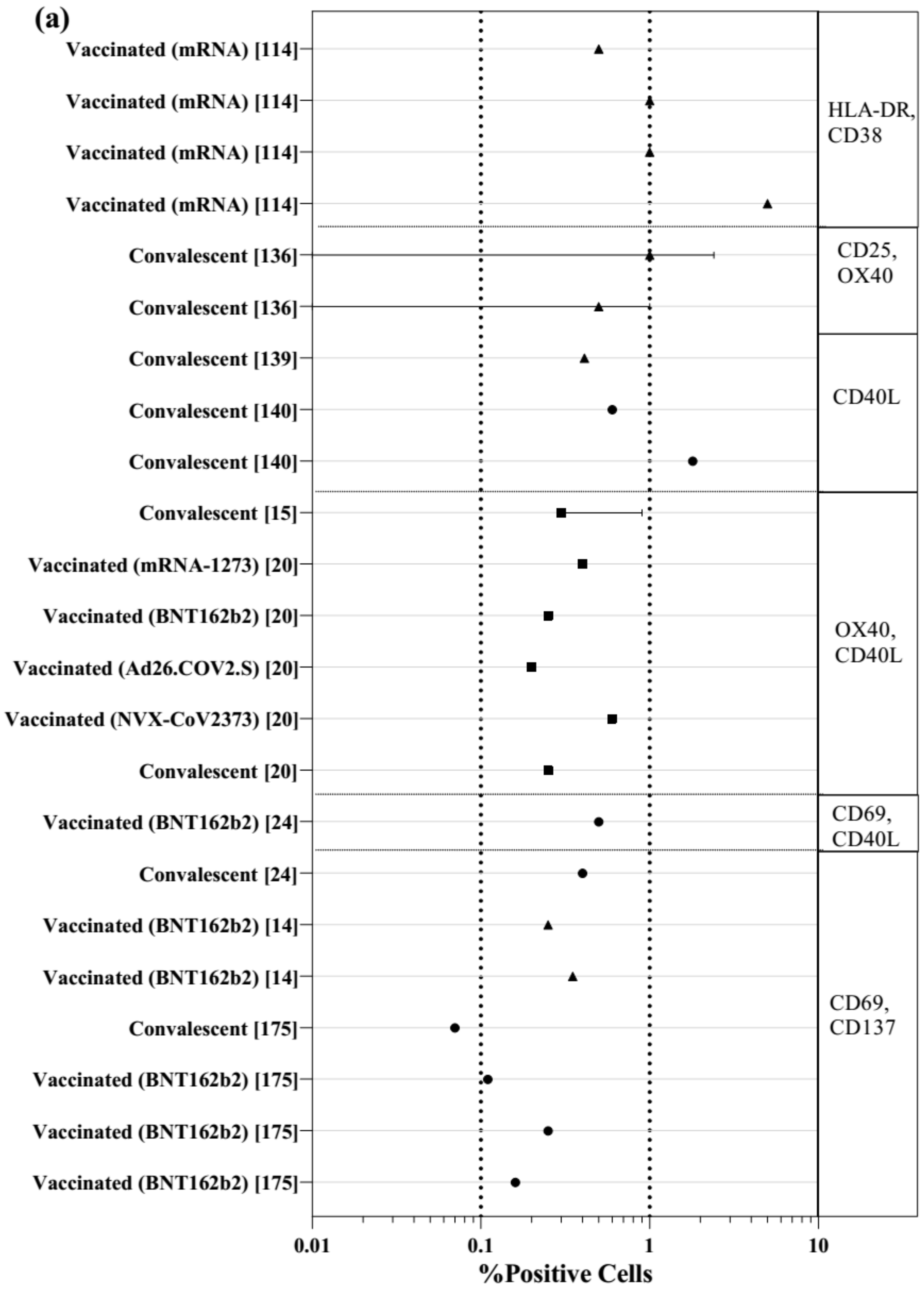

(a)
Vaccinated (mRNA) [114]
Vaccinated (mRNA) [114]
Vaccinated (mRNA) [114]
Vaccinated (mRNA) [114]
Convalescent [136]
Convalescent [136]
Convalescent [139]
Convalescent [140]
Convalescent [140]
Convalescent [15]
Vaccinated (mRNA-1273) [20]
Vaccinated (BNT162b2) [20]
Vaccinated (Ad26.COV2.S) [20]
Vaccinated (NVX-CoV2373) [20]
Convalescent [20]
Vaccinated (BNT162b2) [24]
Convalescent [24]
Vaccinated (BNT162b2) [14]
Vaccinated (BNT162b2) [14]
Convalescent [175]
Vaccinated (BNT162b2) [175]
Vaccinated (BNT162b2) [175]
Vaccinated (BNT162b2) [175]
HLA-DR, CD38
CD25, OX40
CD40L
OX40, CD40L
CD69, CD40L
CD69, CD137
0.01
0.1
1
10
%Positive Cells

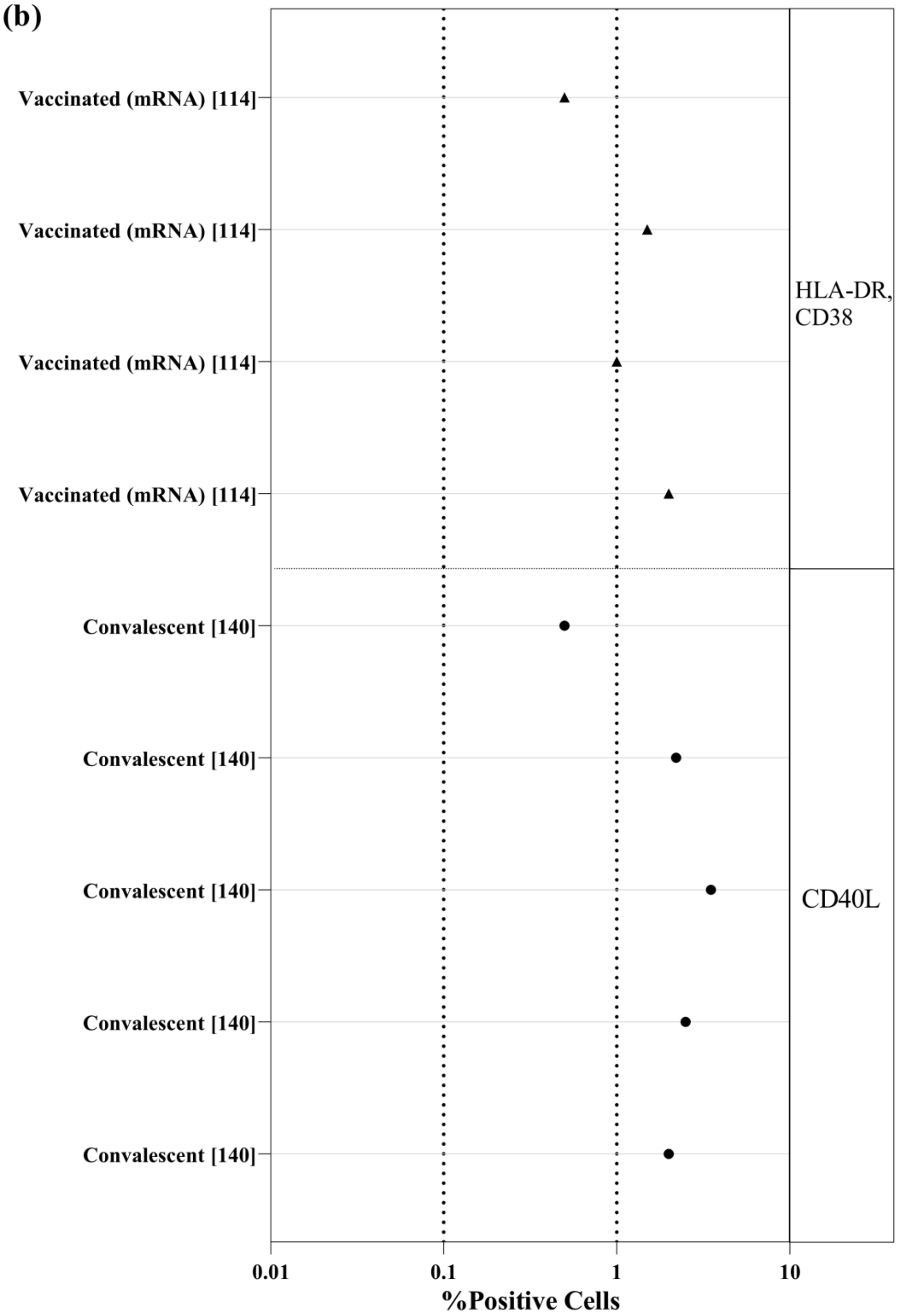

Supplementary Figure 3: Forest plots depicting the range of readouts from AIM assays reported in studies using alternative markers to identify spike-specific T cells for (A) CD4+ and (B) CD8+ cells. For a complete list of descriptive statistics for each study, see **Supplementary Table 5**.

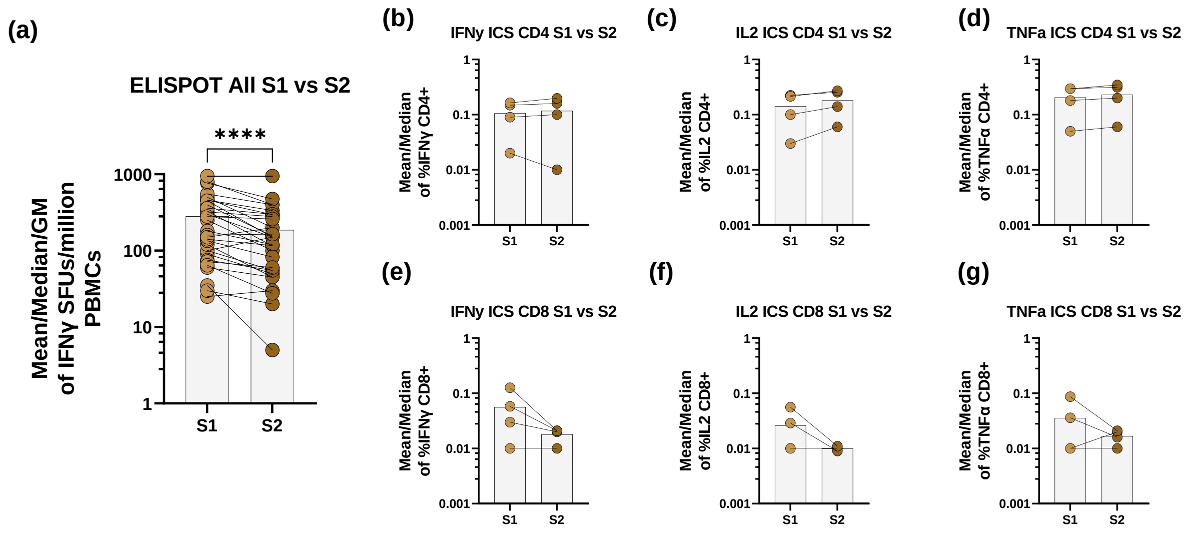

Supplementary Figure 4: Higher antigen-specific T cell response against the S1 domain compared to the S2 domain of SARS-CoV-2 spike protein. Central tendencies reported for the studies that examined T cell responses between the S1 and S2 domains of the spike protein. **(a)** Datasets (n=31) that reported the IFNγ ELISPOT **(b-g)** Datasets (n=4) that reported the intracellular cytokine response. Each dot represents central tendency of one dataset. Lines connect the paired dots of datasets reporting the T cell response between S1 and S2 domains evaluated in same population cohort under similar conditions. Bars represent mean values of the dataset. Wilcoxon-signed rank two-sided paired t-test is performed for statistical analysis. **** $p<0.0001$.

(A)

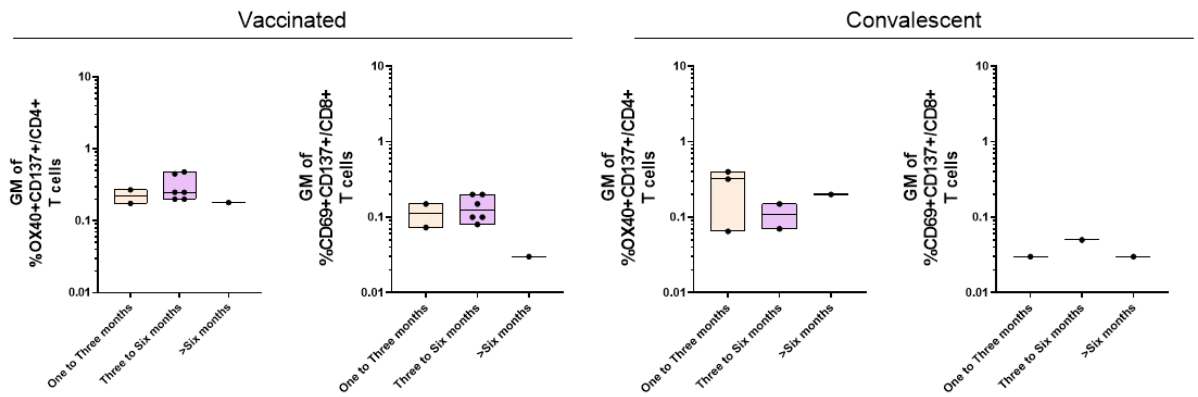

(B)

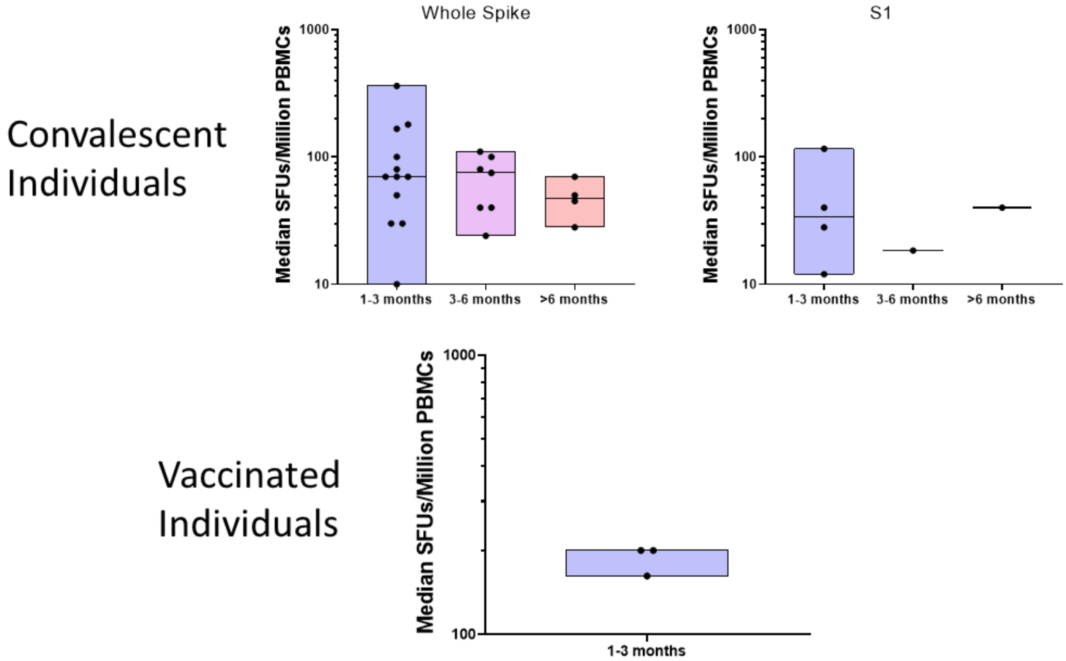

Supplementary Figure 5: The range of spike-specific T cell responses over time according to(A) AIM assays and (B) IFNγ ELISPOT extracted from those studies that reported the duration of time between infection or complete vaccination regimen (WHO EUL vaccines), and sample collection. The graphs depict only those readouts that were provided in Geometric Mean for AIM assays, and Median for IFNγ ELISPOT. Floating bars represent the median minimum and maximum values. For the full list of descriptive statistics, please refer to **Supplementary Table 2** and **S3**.

(a)

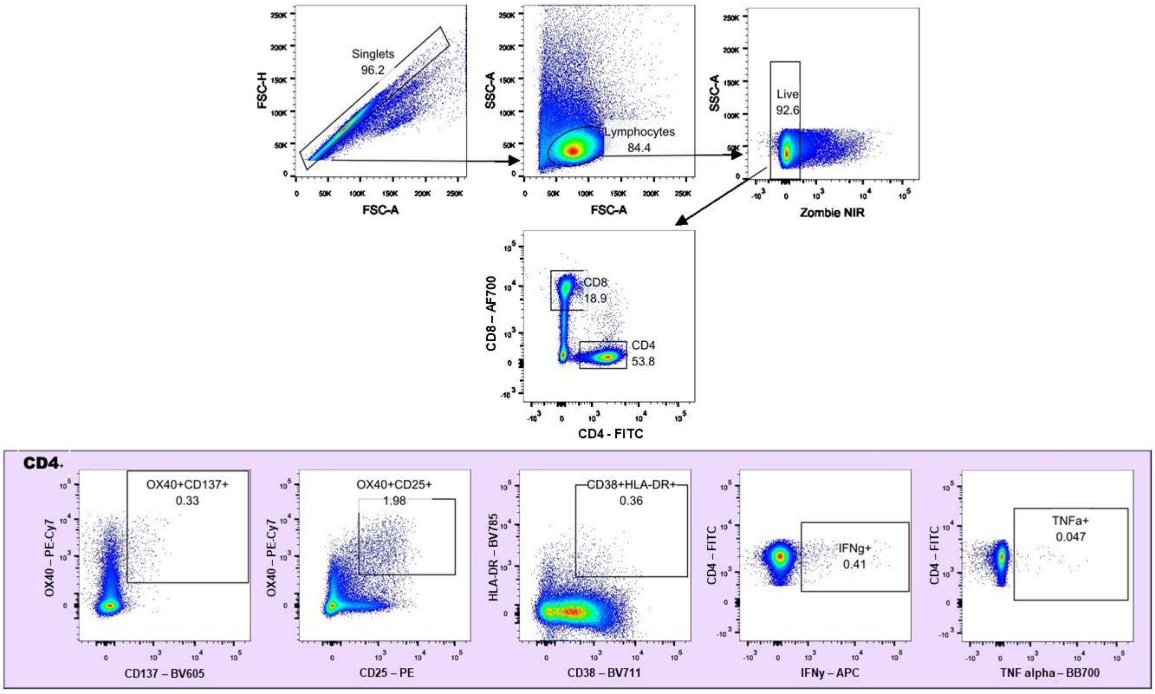

(b)

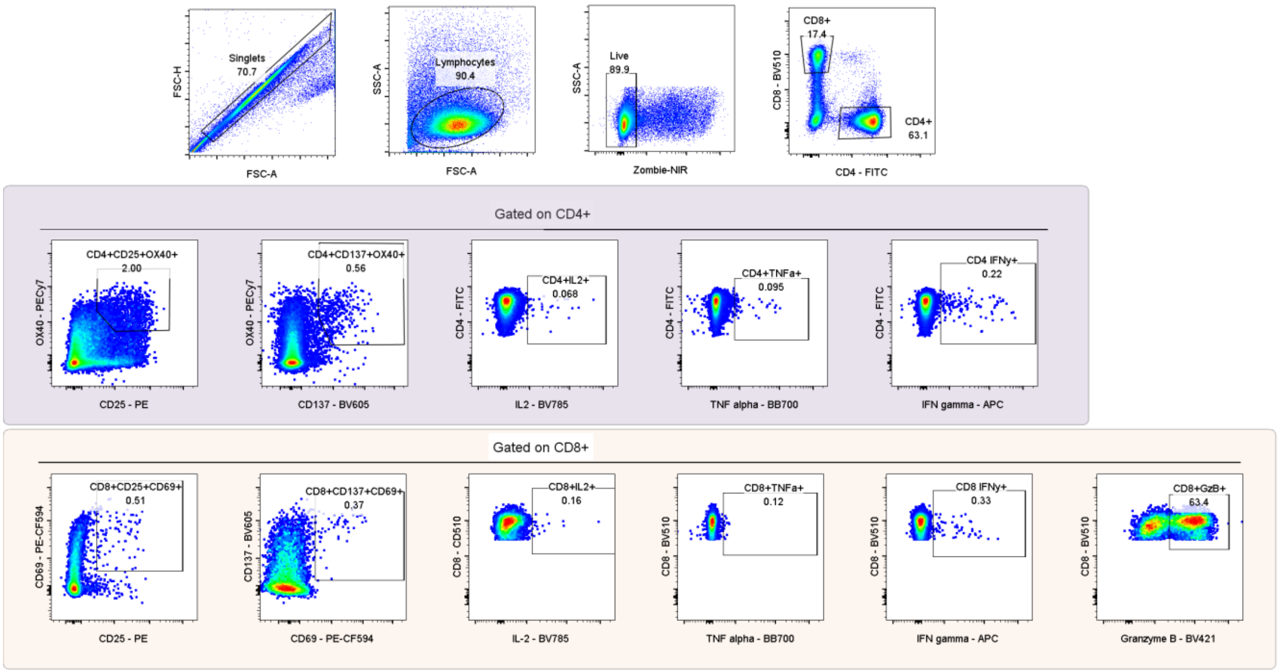

Supplementary Figure 6: **Gating strategy for stimulated PBMCs for validation cohort for AIM and ICS assay.**

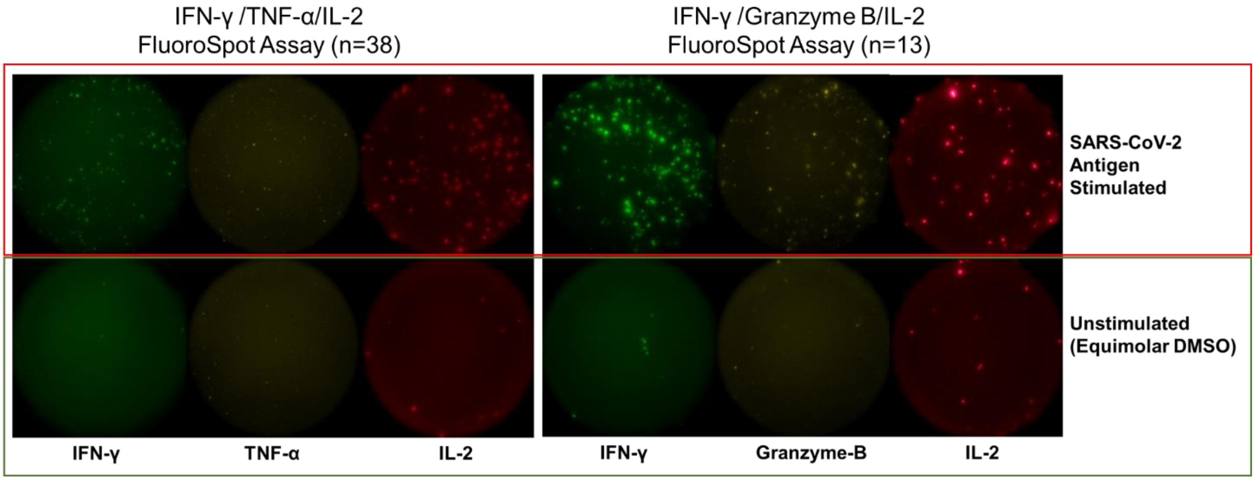

Supplementary Figure 7: Representative figures for the FluoroSpot assay.

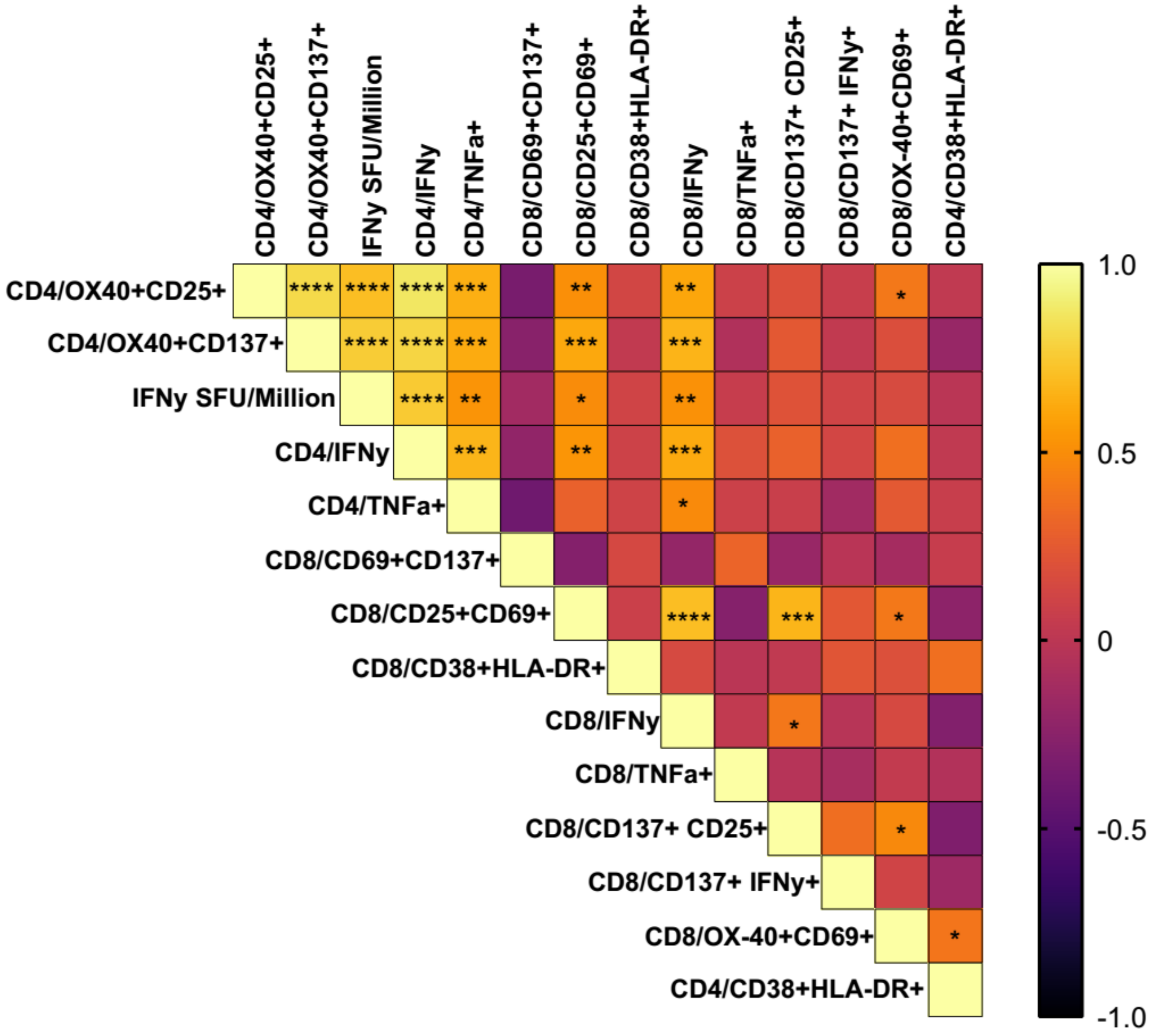

Supplementary Figure 8: Experimental Correlation of different combinations of activation induced markers (AIM) with other antigen-specific T cell assays.

Heatmap depicting the level of correlation between results from different T cell assays simultaneously performed on PBMCs isolated from the same subjects (n=26), based upon Spearman's rank-order correlation. Stars represent p-values, and the intensity of color represents R-values (Spearman's rank correlation coefficient); * $p<0.05$; ** $p<0.01$, *** $p<0.001$, **** $p<0.0001$.

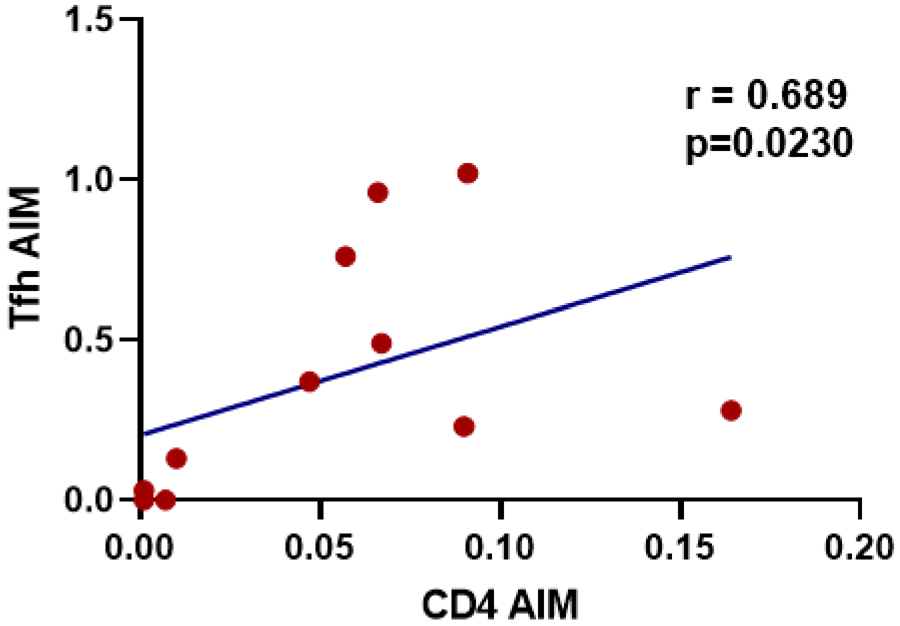

Supplementary Figure 9: Spearman Correlation of the frequencies of CD4 AIM (CD4+ CD137+OX40+) with the frequencies of cTfh AIM (CD3+CD4+CD45RA−CXCR5+ CD40L+ OX40+) in PBMCs of vaccinated individuals (n=11) upon stimulation with whole spike peptide pool.

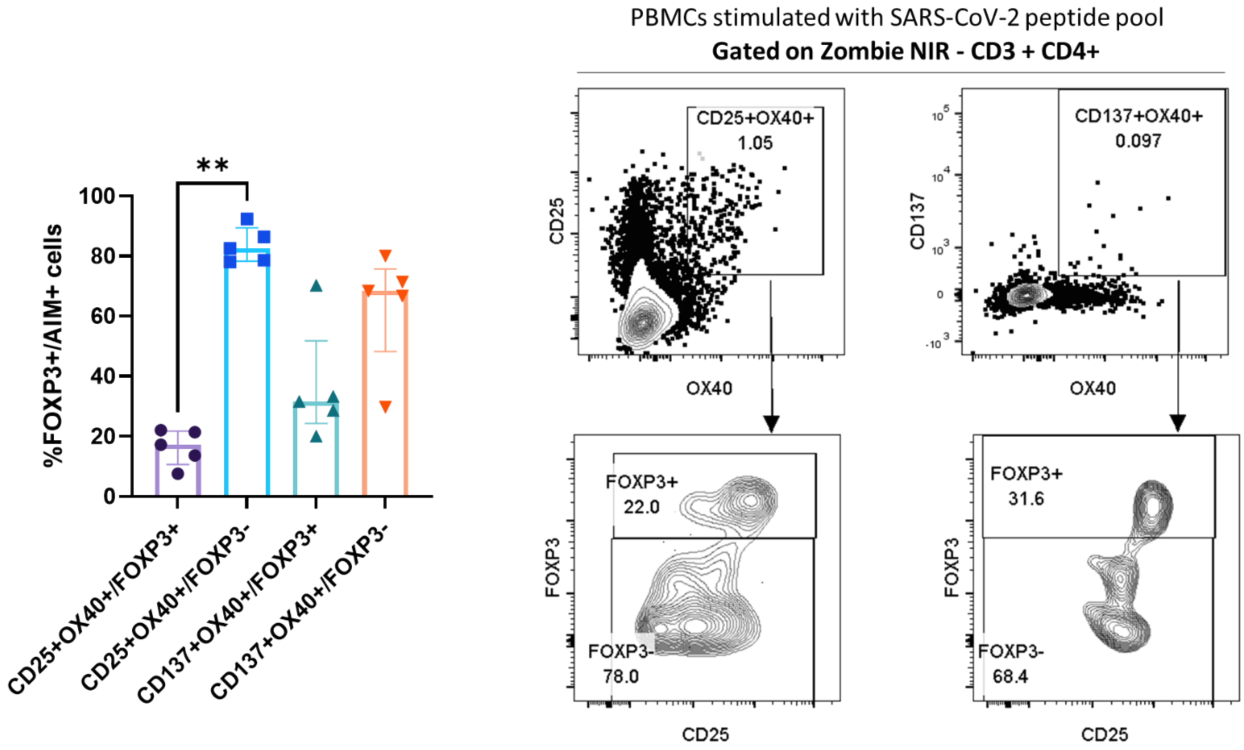

Supplementary Figure 10: Comparison between the frequency of FOXP3+ cells in CD4+CD137+OX40+ cells and CD4+CD25+OX40+ cells upon stimulation of PBMCs (n=5) with SARS-CoV-2 Spike peptide pool for 24h. The CD25+OX40+ cells are predominantly FOXP3 negative and are even higher than the proportion of FOXP3- cells in CD137+OX40+CD4+ cells. Each dot represents data from one individual PBMC sample. Bars represent median %FOXP3/AIM+ cells. Friedman Dunn's multiple comparisons test was performed for statistical analysis. **p<0.01

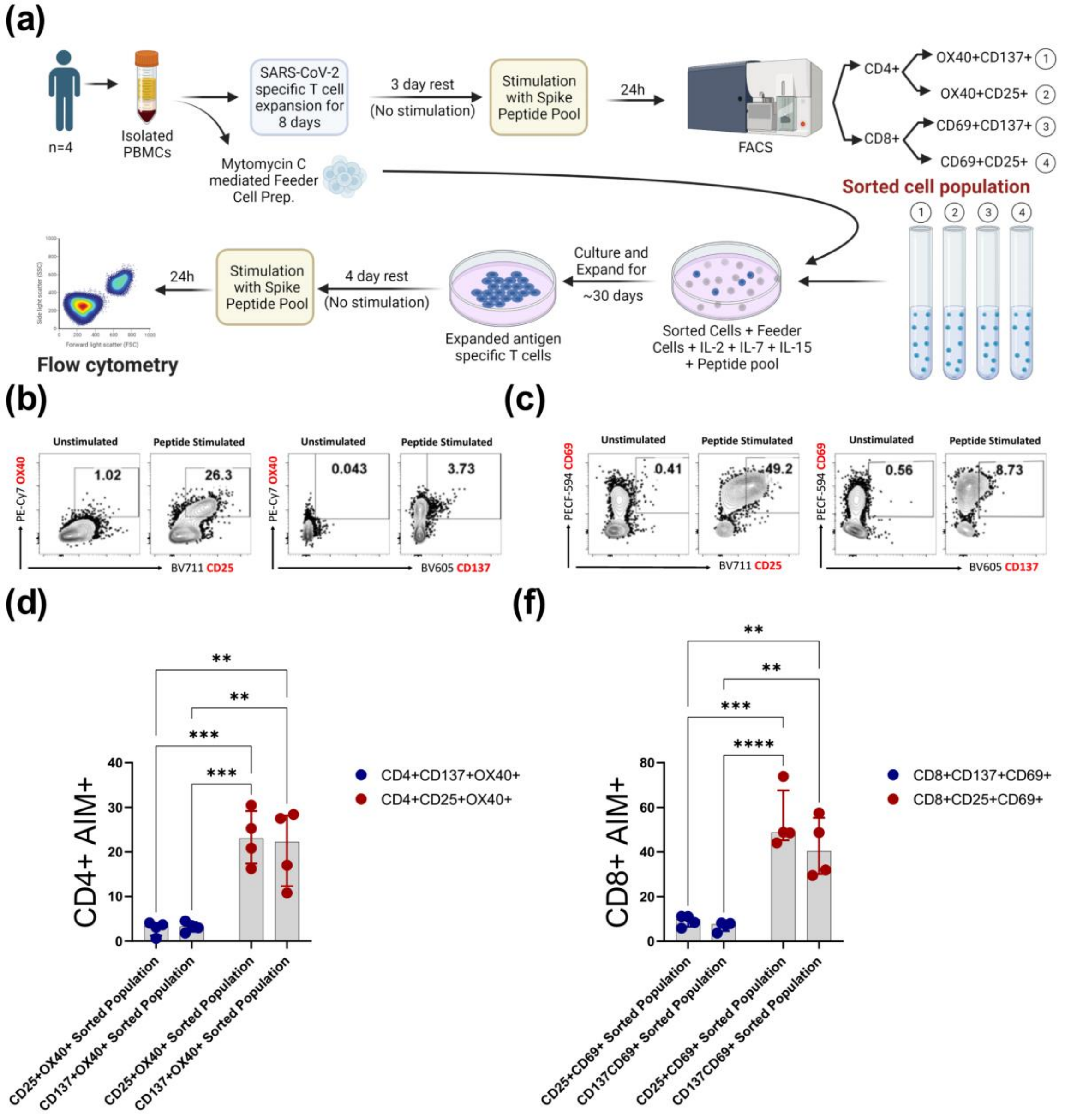

Supplementary Figure 11: Comparison of antigen-specificity of CD137 co-expressing and CD25 co-expressing activation markers by long-term expansion and restimulation. (a) Schematic representation of the experimental design. Briefly, PBMCs (n=4) sorted based on AIM markers were expanded in the presence of antigen followed by restimulation with spike peptide pool and analysis by flow cytometry; created with BioRender.com (b-c) Representative FACS dot plots of the peptide stimulated and unstimulated (DMSO treated) expanded PBMCs of the same sample tested for the expression upon restimulation of (b) OX40/CD25 vs. OX40/CD137 co-expression in CD4 AIM sorted PBMCs (c) CD69/CD25 vs. CD69/CD137 co-expression in CD8 AIM sorted PBMCs (d) Paired comparison of the AIM markers OX40/CD25 (red dots) vs. OX40/CD137 (blue dots) expressed upon

restimulation by the same CD4 AIM sorted PBMC samples (e) Paired comparison of the AIM markers CD69/CD25 (red dots) vs. CD69/CD137 (blue dots) expressed upon restimulation by the CD8 AIM sorted PBMC samples (n=4). Tukey's multiple comparison test was performed for statistical analysis. Each data point shown is background subtracted. Bars and lines represent the median and IQR, respectively. * $p<0.05$; ** $p<0.01$, *** $p<0.001$, **** $p<0.0001$.

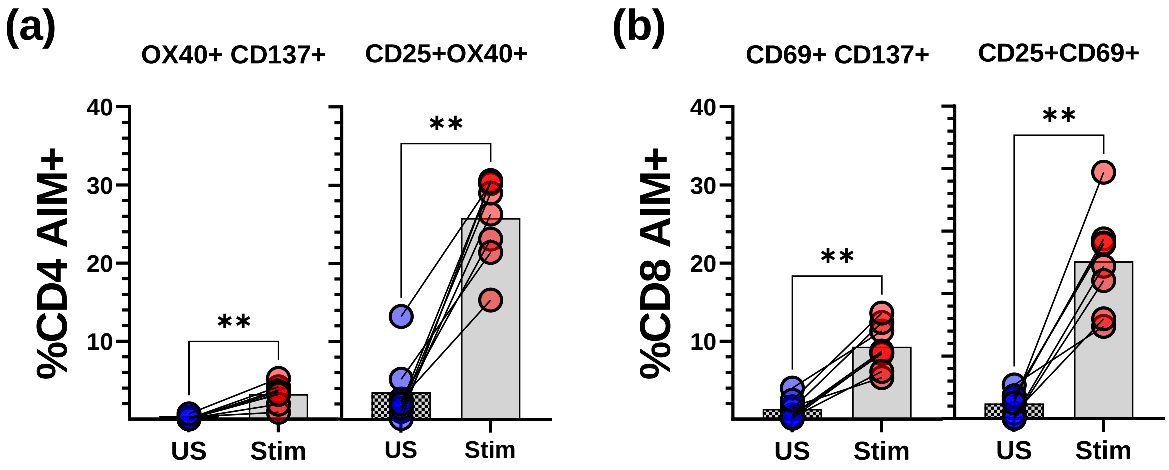

Supplementary Figure 12: Comparison of non-specific background noise while testing the antigen-specificity of CD137 co-expressing and CD25 co-expressing activation markers by long-term expansion and restimulation. Paired comparison of the percentage frequency of AIM markers expressed upon stimulation with peptide pool (red dots) vs unstimulated (equimolar DMSO cultured) (blue dots) in both (a) CD4 and (b) CD8 AIM sorted PBMC samples (n=4). Two-sided Wilcoxon-signed rank t-test was performed for statistical analysis. Bars and lines represent median and IQR respectively.

* p<0.05; ** p<0.01, *** p <0.001, **** p<0.0001.

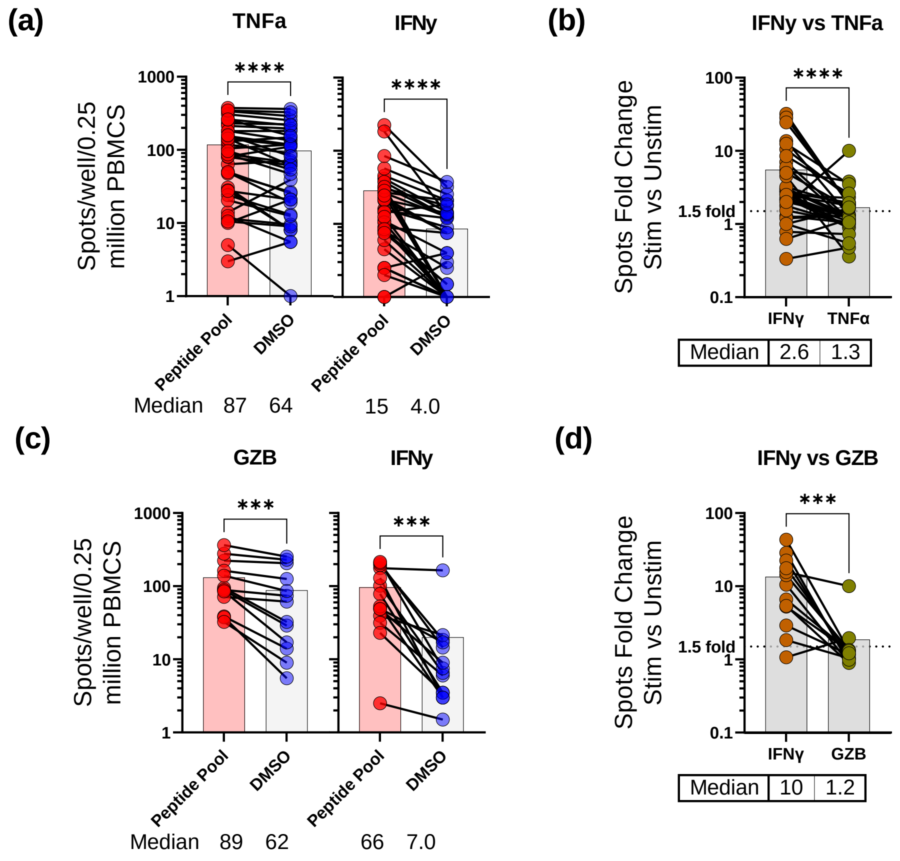

Supplementary Figure 13: Higher non-specific background of TNFα and Granzyme B compared to the IFNγ in FluoroSpot Assay. (a) Comparison between the number of spots formed between peptide stimulated wells and background (DMSO) when tested for TNFα and IFNγ (n=38) and (b) the median fold change of spots observed in stimulated wells compared to the unstimulated wells compared between IFNγ and TNFα. (c) Comparison between the number of spots formed between peptide stimulated wells and background (DMSO) when tested for Granzyme B(GZB) and IFNγ (n=13) and (d) the median fold change of spots observed in stimulated wells compared to the unstimulated wells compared between IFNγ and GZB. Bars represent mean values, and median values are denoted under each bar. Dots represent each sample and the lines connecting the dots connect the paired samples evaluated for IFNγ, TNFα or GZB.

Two-sided Wilcoxon-signed rank t-test was performed for statistical analysis. * $p<0.05$; ** $p<0.01$, *** $p<0.001$, **** $p<0.0001$.

Supplementary Tables:

Supplementary Table 1: Search terms used for PubMed

Keywords used to mine PubMed Database
(sars-cov-2) AND (T-cell) NOT (Review[Publication Type])
(covid-19) AND (T-cell) NOT (Review[Publication Type])

Supplementary Table 2: Summary of the meta-analysis of the %frequencies of spike-specific T cell response determined by the intracellular-cytokine staining (ICS) assay. The number of studies and datasets that examined CD4+ and CD8+ T cells by ICS assays in vaccinated cohorts using different measures of central tendencies, as depicted in Figure 4, and the interquartile range of the central tendencies for each group of datasets. The no. of studies row indicates the number of research articles examining the individual cytokine response in CD4+ and CD8+ T cells and the type of central tendency employed to represent data. The no. of datasets row represents the number of datasets out of the total no. of datasets extracted from various studies for each cohort, as represented in Figure 4, using mean, median, or GM as their measure of central tendency. The range row represents the range of each group of datasets that depicted % of positive cells by mean, median, or GM.

	**Mean**							
	**IFNγ (CD4+)**	**IFNγ (CD8+)**	**IL-2 (CD4+)**	**IL-2 (CD8+)**	**TNFα (CD4+)**	**TNFα (CD8+)**	**IFNγ or IL-2 (CD4+)**	**IFNγ or IL-2 (CD8+)**
No. of studies	5	5	4	3	3	2	-	-
No. of datasets	9 out of 21	9 out of 15	6 out of 18	5 out of 11	5 out of 17	4 out of 10	-	-
Range	0.03%-0.8%	0.02%-0.22%	0.06%-0.271%	0.009%-0.056%	0.295%-0.42%	0.016-0.027	-	-
	**Median**							
No. of studies	5	4	5	4	5	4	1	1
No. of datasets	12 out of 21	6 out of 15	12 out of 18	6 out of 11	12 out of 17	6 out of 10	2 out of 2	2 out of 2
Range	0.01%-0.011%	0.01-0.05%	0.015%-0.14%	0.01%	0.05%-0.35%	0.01%-0.02%	0.08%-0.09%	0.06%-0.07%

Supplementary Table 3: Characteristics of donor cohort

Participant Characteristics

No. of Participants		135
Age (IQR)		34y (26-43)
Gender		
	Female	41
	Male	94
Prior Infection		
	PCR / Antibody confirmed prior infection	47
	No Prior Infection	88
Vaccine Type		
	BBV152	63
	ChAdOx nCoV-19	72
Duration after COVID-19 infection or vaccination - median (IQR)		59 days (32-83)

Supplementary References:

1 Thiruvengadam, R. *et al.* Effectiveness of ChAdOx1 nCoV-19 vaccine against SARS-CoV-2 infection during the delta (B.1.617.2) variant surge in India: a test-negative, case-control study and a mechanistic study of post-vaccination immune responses. *The Lancet Infectious Diseases* **22**, 473-482, doi:10.1016/S1473-3099(21)00680-0 (2022).

2 Le Bert, N. *et al.* SARS-CoV-2-specific T cell immunity in cases of COVID-19 and SARS, and uninfected controls. *Nature* **584**, 457-462 (2020).

3 Ng, O.-W. *et al.* Memory T cell responses targeting the SARS coronavirus persist up to 11 years post-infection. *Vaccine* **34**, 2008-2014 (2016).

4 Kristiansen, P. A. *et al.* WHO International Standard for anti-SARS-CoV-2 immunoglobulin. *The Lancet* **397**, 1347-1348 (2021).

5 McDonald, I., Murray, S. M., Reynolds, C. J., Altmann, D. M. & Boyton, R. J. Comparative systematic review and meta-analysis of reactogenicity, immunogenicity and efficacy of vaccines against SARS-CoV-2. *npj Vaccines* **6**, 74 (2021).

6 Vardhana, S., Baldo, L., Morice, W. G. & Wherry, E. J. Understanding T cell responses to COVID-19 is essential for informing public health strategies. *Science immunology* **7**, eabo1303 (2022).

7 Molodtsov, I. A. *et al.* Severe Acute Respiratory Syndrome Coronavirus 2 (SARS-CoV-2)-Specific T Cells and Antibodies in Coronavirus Disease 2019 (COVID-19) Protection: A Prospective Study. *Clin Infect Dis* **75**, e1-e9, doi:10.1093/cid/ciac278 (2022).

8 Scurr, M. J. *et al.* Magnitude of venous or capillary blood-derived SARS-CoV-2-specific T cell response determines COVID-19 immunity. *Nat Commun* **13**, 5422, doi:10.1038/s41467-022-32985-8 (2022).

9 Almendro-Vázquez, P. *et al.* Cellular and humoral immune responses and breakthrough infections after three SARS-CoV-2 mRNA vaccine doses. *Front Immunol* **13**, 981350, doi:10.3389/fimmu.2022.981350 (2022).

10 Neale, I. *et al.* CD4+ and CD8+ T cells and antibodies are associated with protection against Delta vaccine breakthrough infection: a nested case-control study within the PITCH study. *mBio*, e0121223, doi:10.1128/mbio.01212-23 (2023).

11 Brasu, N. *et al.* Memory CD8(+) T cell diversity and B cell responses correlate with protection against SARS-CoV-2 following mRNA vaccination. *Nat Immunol* **23**, 1445-1456, doi:10.1038/s41590-022-01313-z (2022).

12 Barnes, E. *et al.* SARS-CoV-2-specific immune responses and clinical outcomes after COVID-19 vaccination in patients with immune-suppressive disease. *Nat Med* **29**, 1760-1774, doi:10.1038/s41591-023-02414-4 (2023).

13 Grifoni, A. *et al.* Targets of T cell responses to SARS-CoV-2 coronavirus in humans with COVID-19 disease and unexposed individuals. *Cell* **181**, 1489-1501. e1415 (2020).

14 Geers, D. *et al.* SARS-CoV-2 variants of concern partially escape humoral but not T cell responses in COVID-19 convalescent donors and vaccine recipients. *Science immunology* **6**, eabj1750 (2021).

15 Moderbacher, C. R. *et al.* Antigen-specific adaptive immunity to SARS-CoV-2 in acute COVID-19 and associations with age and disease severity. *Cell* **183**, 996-1012. e1019 (2020).

16 Singh, V. *et al.* Limited induction of SARS-CoV-2–specific T cell responses in children with multisystem inflammatory syndrome compared with COVID-19. *JCI insight* **7** (2022).

17 Jung, J. H. *et al.* SARS-CoV-2-specific T cell memory is sustained in COVID-19 convalescent patients for 10 months with successful development of stem cell-like memory T cells. *Nature communications* **12**, 4043 (2021).

18 Kroemer, M. *et al.* COVID-19 patients display distinct SARS-CoV-2 specific T-cell responses according to disease severity. *Journal of Infection* **82**, 282-327 (2021).

19 Garcia-Valtanen, P. *et al.* SARS-CoV-2 Omicron variant escapes neutralizing antibodies and T cell responses more efficiently than other variants in mild COVID-19 convalescents. *Cell Reports Medicine* **3** (2022).

20 Zhang, Z. *et al.* Humoral and cellular immune memory to four COVID-19 vaccines. *Cell* **185**, 2434-2451. e2417 (2022).

21 Tarke, A. *et al.* Impact of SARS-CoV-2 variants on the total CD4+ and CD8+ T cell reactivity in infected or vaccinated individuals. *Cell Reports Medicine* **2** (2021).

22 Mateus, J. *et al.* Low-dose mRNA-1273 COVID-19 vaccine generates durable memory enhanced by cross-reactive T cells. *Science* **374**, eabj9853 (2021).

23 Kang, C. K. *et al.* Longitudinal analysis of human memory T-cell response according to the severity of illness up to 8 months after severe acute respiratory syndrome coronavirus 2 infection. *The Journal of infectious diseases* **224**, 39-48 (2021).

24 Gao, Y. *et al.* Ancestral SARS-CoV-2-specific T cells cross-recognize the Omicron variant. *Nature medicine* **28**, 472-476 (2022).

25 Sabatino Jr, J. J. *et al.* Multiple sclerosis therapies differentially affect SARS-CoV-2 vaccine–induced antibody and T cell immunity and function. *JCI insight* **7** (2022).

26 Tarke, A. *et al.* SARS-CoV-2 vaccination induces immunological T cell memory able to cross-recognize variants from Alpha to Omicron. *Cell* **185**, 847-859. e811 (2022).

27 Kim, J. Y. *et al.* SARS-CoV-2-specific antibody and T cell response kinetics according to symptom severity. *The American journal of tropical medicine and hygiene* **105**, 395 (2021).

28 Chen, G.-L. *et al.* Safety and immunogenicity of the SARS-CoV-2 ARCoV mRNA vaccine in Chinese adults: a randomised, double-blind, placebo-controlled, phase 1 trial. *The Lancet Microbe* **3**, e193-e202 (2022).

29 Sahin, U. *et al.* COVID-19 vaccine BNT162b1 elicits human antibody and TH1 T cell responses. *Nature* **586**, 594-599 (2020).

30 Sahin, U. *et al.* BNT162b2 vaccine induces neutralizing antibodies and poly-specific T cells in humans. *Nature* **595**, 572-577 (2021).

31 Vályi-Nagy, I. *et al.* Comparison of antibody and T cell responses elicited by BBIBP-CorV (Sinopharm) and BNT162b2 (Pfizer-BioNTech) vaccines against SARS-CoV-2 in healthy adult humans. *Geroscience* **43**, 2321-2331 (2021).

32 Cohen, H. *et al.* T cell response following anti-COVID-19 BNT162b2 vaccination is maintained against the SARS-CoV-2 Omicron B. 1.1. 529 variant of concern. *Viruses* **14**, 347 (2022).

33 Prendecki, M. *et al.* Effect of previous SARS-CoV-2 infection on humoral and T-cell responses to single-dose BNT162b2 vaccine. *The Lancet* **397**, 1178-1181 (2021).

34 Angyal, A. *et al.* T-cell and antibody responses to first BNT162b2 vaccine dose in previously infected and SARS-CoV-2-naive UK health-care workers: a multicentre prospective cohort study. *The Lancet Microbe* **3**, e21-e31 (2022).

35 Demaret, J. *et al.* Impaired functional T-cell response to SARS-CoV-2 after two doses of BNT162b2 mRNA vaccine in older people. *Frontiers in immunology* **12**, 778679 (2021).

36 Parry, H. *et al.* Immunogenicity of single vaccination with BNT162b2 or ChAdOx1 nCoV-19 at 5–6 weeks post vaccine in participants aged 80 years or older: an exploratory analysis. *The Lancet Healthy Longevity* **2**, e554-e560 (2021).

37 Thümmler, L. *et al.* Comparison of SARS-CoV-2-and HCoV-specific T cell response using IFN-γ ELISpot. *Diagnostics* **11**, 1439 (2021).

38 Munro, A. P. *et al.* Safety and immunogenicity of seven COVID-19 vaccines as a third dose (booster) following two doses of ChAdOx1 nCov-19 or BNT162b2 in the UK (COV-BOOST): a blinded, multicentre, randomised, controlled, phase 2 trial. *The Lancet* **398**, 2258-2276 (2021).

39 Ramasamy, M. N. *et al.* Safety and immunogenicity of ChAdOx1 nCoV-19 vaccine administered in a prime-boost regimen in young and old adults (COV002): a single-blind, randomised, controlled, phase 2/3 trial. *The Lancet* **396**, 1979-1993 (2020).

40 Stuart, A. S. *et al.* Immunogenicity, safety, and reactogenicity of heterologous COVID-19 primary vaccination incorporating mRNA, viral-vector, and protein-adjuvant vaccines in the UK (Com-COV2): a single-blind, randomised, phase 2, non-inferiority trial. *The Lancet* **399**, 36-49 (2022).

41 Folegatti, P. M. *et al.* Safety and immunogenicity of the ChAdOx1 nCoV-19 vaccine against SARS-CoV-2: a preliminary report of a phase 1/2, single-blind, randomised controlled trial. *The Lancet* **396**, 467-478 (2020).

42 Jeewandara, C. *et al.* Immune responses to a single dose of the AZD1222/Covishield vaccine at 16 weeks in individuals in Sri Lanka. *The Journal of Immunology* **207**, 2681-2687 (2021).

43 Jeewandara, C. *et al.* Kinetics of immune responses to the AZD1222/Covishield vaccine with varying dose intervals in Sri Lankan individuals. *Immunity, inflammation and disease* **10**, e592 (2022).

44 Jeewandara, C. *et al.* Immune responses to a single dose of the AZD1222/Covishield vaccine in health care workers. *Nature Communications* **12**, 4617, doi:10.1038/s41467-021-24579-7 (2021).

45 Stephenson, K. E. *et al.* Immunogenicity of the Ad26. COV2. S Vaccine for COVID-19. *Jama* **325**, 1535-1544 (2021).

46 Alter, G. *et al.* Immunogenicity of Ad26. COV2. S vaccine against SARS-CoV-2 variants in humans. *Nature* **596**, 268-272 (2021).

47 Zhu, F.-C. *et al.* Immunogenicity and safety of a recombinant adenovirus type-5-vectored COVID-19 vaccine in healthy adults aged 18 years or older: a randomised, double-blind, placebo-controlled, phase 2 trial. *The Lancet* **396**, 479-488 (2020).

48 Zhu, F.-C. *et al.* Safety, tolerability, and immunogenicity of a recombinant adenovirus type-5 vectored COVID-19 vaccine: a dose-escalation, open-label, non-randomised, first-in-human trial. *The Lancet* **395**, 1845-1854 (2020).

49 Escobar, A. *et al.* Evaluation of the immune response induced by CoronaVac 28-day schedule vaccination in a healthy population group. *Frontiers in immunology* **12**, 766278 (2022).

50 Melo-González, F. *et al.* Recognition of variants of concern by antibodies and T cells induced by a SARS-CoV-2 inactivated vaccine. *Frontiers in immunology* **12**, 747830 (2021).

51 Jeewandara, C. *et al.* Persistence of immune responses to the Sinopharm/BBIBP-CorV vaccine. *Immunity, inflammation and disease* **10**, e621 (2022).

52 Ella, R. *et al.* Safety and immunogenicity of an inactivated SARS-CoV-2 vaccine, BBV152: a double-blind, randomised, phase 1 trial. *The Lancet Infectious Diseases* **21**, 637-646 (2021).

53 Tebas, P. *et al.* Safety and immunogenicity of INO-4800 DNA vaccine against SARS-CoV-2: A preliminary report of an open-label, Phase 1 clinical trial. *EClinicalMedicine* **31** (2021).

54 Prasithsirikul, W. *et al.* Immunogenicity of ChAdOx1 nCoV-19 booster vaccination following two CoronaVac shots in healthcare workers. *Vaccines* **10**, 217 (2022).

55 Woldemeskel, B. A., Garliss, C. C. & Blankson, J. N. SARS-CoV-2 mRNA vaccines induce broad CD4+ T cell responses that recognize SARS-CoV-2 variants and HCoV-NL63. *The Journal of clinical investigation* **131** (2021).

56 Ogbe, A. *et al.* T cell assays differentiate clinical and subclinical SARS-CoV-2 infections from cross-reactive antiviral responses. *Nature Communications* **12**, 2055, doi:10.1038/s41467-021-21856-3 (2021).

57 Le Bert, N. *et al.* Highly functional virus-specific cellular immune response in asymptomatic SARS-CoV-2 infection. *Journal of Experimental Medicine* **218**, e20202617 (2021).

58 Woldemeskel, B. A. *et al.* Healthy donor T cell responses to common cold coronaviruses and SARS-CoV-2. *The Journal of clinical investigation* **130**, 6631-6638 (2020).

59 Kruse, M. *et al.* Performance of the T-SPOT®. COVID test for detecting SARS-CoV-2-responsive T cells. *International Journal of Infectious Diseases* **113**, 155-161 (2021).

60 Yang, J. *et al.* Characteristics of T-cell responses in COVID-19 patients with prolonged SARS-CoV-2 positivity–a cohort study. *Clinical & Translational Immunology* **10**, e1259 (2021).

61 Zuo, J. *et al.* Robust SARS-CoV-2-specific T cell immunity is maintained at 6 months following primary infection. *Nature immunology* **22**, 620-626 (2021).

62 Gurevich, M. *et al.* SARS-CoV-2 memory B and T cell profiles in mild COVID-19 convalescent patients. *International Journal of Infectious Diseases* **115**, 208-214 (2022).

63 Schwarzkopf, S. *et al.* Cellular immunity in COVID-19 convalescents with PCR-confirmed infection but with undetectable SARS-CoV-2–specific IgG. *Emerging infectious diseases* **27**, 122 (2021).

64 Kim, N. *et al.* Off-the-shelf partial HLA matching SARS-CoV-2 antigen specific T cell therapy: a new possibility for COVID-19 treatment. *Frontiers in Immunology* **12**, 751869 (2021).

65 Björkander, S. *et al.* SARS-CoV-2–specific B-and T-cell immunity in a population-based study of young Swedish adults. *Journal of Allergy and Clinical Immunology* **149**, 65-75. e68 (2022).

66 Lindemann, M. *et al.* Humoral and cellular vaccination responses against SARS-CoV-2 in hematopoietic stem cell transplant recipients. *Vaccines* **9**, 1075 (2021).

67 Jackson, L. A. *et al.* An mRNA vaccine against SARS-CoV-2—preliminary report. *New England journal of medicine* **383**, 1920-1931 (2020).

68 Anderson, E. J. *et al.* Safety and immunogenicity of SARS-CoV-2 mRNA-1273 vaccine in older adults. *New England Journal of Medicine* **383**, 2427-2438 (2020).

69 Swanson, P. A. *et al.* AZD1222/ChAdOx1 nCoV-19 vaccination induces a polyfunctional spike protein–specific TH1 response with a diverse TCR repertoire. *Science translational medicine* **13**, eabj7211 (2021).

70 Ewer, K. J. *et al.* T cell and antibody responses induced by a single dose of ChAdOx1 nCoV-19 (AZD1222) vaccine in a phase 1/2 clinical trial. *Nature medicine* **27**, 270-278 (2021).

71 Sadoff, J. *et al.* Interim results of a phase 1–2a trial of Ad26. COV2. S Covid-19 vaccine. *New England Journal of Medicine* **384**, 1824-1835 (2021).

72 Seddiki, N. *et al.* Human antigen-specific CD4+ CD25+ CD134+ CD39+ T cells are enriched for regulatory T cells and comprise a substantial proportion of recall responses. *European journal of immunology* **44**, 1644-1661 (2014).

73 Petrone, L. *et al.* A whole blood test to measure SARS-CoV-2-specific response in COVID-19 patients. *Clinical Microbiology and Infection* **27**, 286.e287-286.e213, doi:https://doi.org/10.1016/j.cmi.2020.09.051 (2021).

74 Martínez-Gallo, M. *et al.* Commercialized kits to assess T-cell responses against SARS-CoV-2 S peptides. A pilot study in health care workers. *Medicina Clinica* **159**, 116-123 (2022).

75 Fernández-González, M. *et al.* Clinical Performance of a Standardized Severe Acute Respiratory Syndrome Coronavirus 2 (SARS-CoV-2) Interferon-γ Release Assay for Simple Detection of T-Cell Responses After Infection or Vaccination. *Clin Infect Dis* **75**, e338-e346, doi:10.1093/cid/ciab1021 (2022).

76 Fong, C. H.-Y. *et al.* Effect of vaccine booster, vaccine type, and hybrid immunity on humoral and cellular immunity against SARS-CoV-2 ancestral strain and Omicron variant sublineages BA. 2 and BA. 5 among older adults with comorbidities: A cross sectional study. *EBioMedicine* **88** (2023).

77 Scurr, M. J. *et al.* Whole blood-based measurement of SARS-CoV-2-specific T cells reveals asymptomatic infection and vaccine immunogenicity in healthy subjects and patients with solid-organ cancers. *Immunology* **165**, 250-259 (2022).

78 Adetifa, I. M. *et al.* Comparison of two interferon gamma release assays in the diagnosis of Mycobacterium tuberculosis infection and disease in The Gambia. *BMC Infect Dis* **7**, 122, doi:10.1186/1471-2334-7-122 (2007).

79 Johnson, S. A. *et al.* Evaluation of QuantiFERON SARS-CoV-2 interferon-γ release assay following SARS-CoV-2 infection and vaccination. *Clin Exp Immunol* **212**, 249-261, doi:10.1093/cei/uxad027 (2023).

80 de Vries, R. D., van der Heiden, M., Geers, D., Imhof, C. & van Baarle, D. Difference in sensitivity between SARS-CoV-2–specific T cell assays in patients with underlying conditions. *The Journal of Clinical Investigation* **131**, doi:10.1172/JCI155499 (2021).

81 Schwarz, M. *et al.* Rapid, scalable assessment of SARS-CoV-2 cellular immunity by whole-blood PCR. *Nature Biotechnology* **40**, 1680-1689, doi:10.1038/s41587-022-01347-6 (2022).

82 Reiss, S. *et al.* Comparative analysis of activation induced marker (AIM) assays for sensitive identification of antigen-specific CD4 T cells. *PloS one* **12**, e0186998 (2017).

83 Seddiki, N. *et al.* Expression of interleukin (IL)-2 and IL-7 receptors discriminates between human regulatory and activated T cells. *The Journal of experimental medicine* **203**, 1693-1700 (2006).

84 Zaunders, J. J. *et al.* High levels of human antigen-specific CD4+ T cells in peripheral blood revealed by stimulated coexpression of CD25 and CD134 (OX40). *The Journal of Immunology* **183**, 2827-2836 (2009).

85 Lehmann, P. V. *et al.* Comprehensive evaluation of the expressed CD8+ T cell epitope space using high-throughput epitope mapping. *Frontiers in Immunology* **10**, 655 (2019).

86 Poloni, C. *et al.* T-cell activation–induced marker assays in health and disease. *Immunology and Cell Biology* (2023).

87 Sommer, U. *et al.* High-sensitivity flow cytometric assays: Considerations for design control and analytical validation for identification of Rare events. *Cytometry Part B: Clinical Cytometry* **100**, 42-51, doi:https://doi.org/10.1002/cyto.b.21949 (2021).

88 Hedley, B. & Keeney, M. Technical issues: flow cytometry and rare event analysis. *International journal of laboratory hematology* **35**, 344-350 (2013).

89 Sahin, U. *et al.* COVID-19 vaccine BNT162b1 elicits human antibody and TH1 T cell responses. *Nature* **586**, 594-599, doi:10.1038/s41586-020-2814-7 (2020).

90 Binayke, A. *et al.* Proinflammatory Innate Cytokines and Distinct Metabolomic Signatures Shape the T Cell Response in Active COVID-19. *Vaccines* **10**, 1762 (2022).

91 Mok, C. K. P. *et al.* Omicron BA. 1-specific T-cell responses in adults vaccinated with CoronaVac or BNT162b2 in Hong Kong: an observational cohort study. *The Lancet Microbe* **4**, e418-e430 (2023).

92 Altosole, T., Rotta, G., Uras, C. R. M., Bornheimer, S. J. & Fenoglio, D. An optimized flow cytometry protocol for simultaneous detection of T cell activation induced markers and intracellular cytokines: Application to SARS-CoV-2 immune individuals. *Journal of Immunological Methods* **515**, 113443, doi:https://doi.org/10.1016/j.jim.2023.113443 (2023).

93 Zhang, Z. *et al.* Humoral and cellular immune memory to four COVID-19 vaccines. *Cell* **185**, 2434-2451.e2417, doi:https://doi.org/10.1016/j.cell.2022.05.022 (2022).

94 Lehmann, P. V. *et al.* Comprehensive Evaluation of the Expressed CD8+ T Cell Epitope Space Using High-Throughput Epitope Mapping. *Front Immunol* **10**, 655, doi:10.3389/fimmu.2019.00655 (2019).

95 Zhang, W., Moldovan, I., Targoni, O. S., Subbramanian, R. A. & Lehmann, P. V. How much of virus-specific CD8 T cell reactivity is detected with a peptide pool when compared to individual peptides? *Viruses* **4**, 2636-2649, doi:10.3390/v4112636 (2012).

96 Poluektov, Y., George, M., Daftarian, P. & Delcommenne, M. C. Assessment of SARS-CoV-2 specific CD4 (+) and CD8 (+) T cell responses using MHC class I and II tetramers. *Vaccine* **39**, 2110-2116 (2021).

97 Peng, Y. *et al.* Broad and strong memory CD4+ and CD8+ T cells induced by SARS-CoV-2 in UK convalescent individuals following COVID-19. *Nature immunology* **21**, 1336-1345 (2020).

98 Gao, F. *et al.* Spheromers reveal robust T cell responses to the Pfizer/BioNTech vaccine and attenuated peripheral CD8+ T cell responses post SARS-CoV-2 infection. *Immunity* **56**, 864-878. e864 (2023).

99 Shoukat, M. S. *et al.* Use of machine learning to identify a T cell response to SARS-CoV-2. *Cell Reports Medicine* **2** (2021).

100 Ameratunga, R. *et al.* Perspective: diagnostic laboratories should urgently develop T cell assays for SARS-CoV-2 infection. *Expert review of clinical immunology* **17**, 421-430 (2021).

101 Bowyer, G. *et al.* Activation-induced Markers Detect Vaccine-Specific CD4+ T Cell Responses Not Measured by Assays Conventionally Used in Clinical Trials. *Vaccines* **6**, 50 (2018).

102 Villemonteix, J. *et al.* Comparison between enzyme-linked immunospot assay and intracellular cytokine flow cytometry assays for the evaluation of T cell response to SARS-CoV-2 after symptomatic COVID-19. *Immun Inflamm Dis* **10**, e617, doi:10.1002/iid3.617 (2022).

103 Tassignon, J. *et al.* Monitoring of cellular responses after vaccination against tetanus toxoid: comparison of the measurement of IFN-γ production by ELISA, ELISPOT, flow cytometry and real-time PCR. *Journal of immunological methods* **305**, 188-198 (2005).

104 Meierhoff, G., Ott, P. A., Lehmann, P. V. & Schloot, N. C. Cytokine detection by ELISPOT: relevance for immunological studies in type 1 diabetes. *Diabetes/metabolism research and reviews* **18**, 367-380 (2002).

105 Mateus, J. *et al.* Selective and cross-reactive SARS-CoV-2 T cell epitopes in unexposed humans. *Science* **370**, 89-94 (2020).

106 Thuluva, S. *et al.* Safety, tolerability and immunogenicity of Biological E's CORBEVAX™ vaccine in children and adolescents: A prospective, randomised, double-blind, placebo controlled, phase-2/3 study. *Vaccine* **40**, 7130-7140 (2022).

107 Binayke, A. *et al.* Proinflammatory innate cytokines and distinct metabolomic signatures shape the T cell response in active COVID-19. *Vaccines* **10**, 1762 (2022).

108 Bozkus, C. C., Blazquez, A. B., Enokida, T. & Bhardwaj, N. A T-cell-based immunogenicity protocol for evaluating human antigen-specific responses. *STAR protocols* **2**, 100758 (2021).

109 Zavaglio, F. *et al.* Robust and persistent b-and t-cell responses after covid-19 in immunocompetent and solid organ transplant recipient patients. *Viruses* **13**, 2261 (2021).

110 Ogbe, A. *et al.* Durability of ChAdOx1 nCoV-19 vaccination in people living with HIV. *JCI insight* **7** (2022).

111 Sauerwein, K. M. *et al.* Antigen-specific CD4+ T-cell activation in primary antibody deficiency after BNT162b2 mRNA COVID-19 vaccination. *Frontiers in immunology* **13**, 827048 (2022).

112 Rank, A. *et al.* One year after mild COVID-19: the majority of patients maintain specific immunity, but one in four still suffer from long-term symptoms. *Journal of clinical medicine* **10**, 3305 (2021).

113 Dennehy, K. M. *et al.* Comparison of the development of SARS-coronavirus-2-specific cellular immunity, and central memory CD4+ T-cell responses following Infection versus vaccination. *Vaccines* **9**, 1439 (2021).

114 Lucas, C. *et al.* Impact of circulating SARS-CoV-2 variants on mRNA vaccine-induced immunity. *Nature* **600**, 523-529 (2021).

115 Loyal, L. *et al.* Cross-reactive CD4+ T cells enhance SARS-CoV-2 immune responses upon infection and vaccination. *Science* **374**, eabh1823 (2021).

116 Painter, M. M. *et al.* Rapid induction of antigen-specific CD4+ T cells is associated with coordinated humoral and cellular immunity to SARS-CoV-2 mRNA vaccination. *Immunity* **54**, 2133-2142. e2133 (2021).

117 Tan, A. T. *et al.* Rapid measurement of SARS-CoV-2 spike T cells in whole blood from vaccinated and naturally infected individuals. *The Journal of clinical investigation* **131** (2021).

118 Law, J. C. *et al.* Persistence of T cell and antibody responses to SARS-CoV-2 up to 9 months after symptom onset. *The Journal of Immunology* **208**, 429-443 (2022).

119 Wang, D. *et al.* Transcriptomic characteristics and impaired immune function of patients who retest positive for SARS-CoV-2 RNA. *Journal of Molecular Cell Biology* **13**, 748-759 (2021).

120 Yang, X. *et al.* Naturally activated adaptive immunity in COVID-19 patients. *Journal of cellular and molecular medicine* **24**, 12457-12463 (2020).

121 Tan, H. X. *et al.* Adaptive immunity to human coronaviruses is widespread but low in magnitude. *Clinical & translational immunology* **10**, e1264 (2021).

122 Gil-Manso, S. *et al.* Comprehensive flow cytometry profiling of the immune system in COVID-19 convalescent individuals. *Frontiers in immunology* **12**, 793142 (2022).

123 Anft, M. *et al.* COVID-19-induced ARDS is associated with decreased frequency of activated memory/effector T cells expressing CD11a++. *Molecular Therapy* **28**, 2691-2702 (2020).

124 Moga, E., Lynton-Pons, E. & Domingo, P. The robustness of cellular immunity determines the fate of SARS-CoV-2 infection. *Frontiers in Immunology* **13**, 904686 (2022).

125 Kremer, A. E. *et al.* Successful treatment of COVID-19 infection with convalescent plasma in B-cell-depleted patients may promote cellular immunity. *European journal of immunology* **51**, 2478-2484 (2021).

126 Varchetta, S. *et al.* Unique immunological profile in patients with COVID-19. *Cellular & molecular immunology* **18**, 604-612 (2021).

127 Files, J. K. *et al.* Sustained cellular immune dysregulation in individuals recovering from SARS-CoV-2 infection. *The Journal of Clinical Investigation* **131** (2021).

128 Sureshchandra, S. *et al.* Single-cell RNA sequencing reveals immunological rewiring at the maternal-fetal interface following asymptomatic/mild SARS-CoV-2 infection. *Cell Rep* **39**, 110938, doi:10.1016/j.celrep.2022.110938 (2022).

129 Hennings, V. *et al.* The presence of serum anti-SARS-CoV-2 IgA appears to protect primary health care workers from COVID-19. *European Journal of Immunology* **52**, 800-809 (2022).

130 Herrmann, M. *et al.* Analysis of co-inhibitory receptor expression in COVID-19 infection compared to acute plasmodium falciparum malaria: lAG-3 and TIM-3 correlate with t cell activation and course of disease. *Frontiers in immunology* **11**, 1870 (2020).

131 Pierce, C. A. *et al.* Immune responses to SARS-CoV-2 infection in hospitalized pediatric and adult patients. *Science translational medicine* **12**, eabd5487 (2020).

132 Kratzer, B. *et al.* Immunological imprint of COVID-19 on human peripheral blood leukocyte populations. *Allergy* **76**, 751-765 (2021).

133 Kalicińska, E. *et al.* Lymphocyte subsets in haematological patients with COVID-19: Multicentre prospective study. *Translational Oncology* **14**, 100943 (2021).

134 San Segundo, D. *et al.* Immune assessment of BNT162b2 m-RNA-spike based vaccine response in adults. *Biomedicines* **9**, 868 (2021).

135 Bolouri, H. *et al.* The COVID-19 immune landscape is dynamically and reversibly correlated with disease severity. *The Journal of Clinical Investigation* **131** (2021).

136 Balachandran, H. *et al.* Maintenance of broad neutralizing antibodies and memory B cells 1 year post-infection is predicted by SARS-CoV-2-specific CD4+ T cell responses. *Cell reports* **38** (2022).

137 Tang, G. *et al.* The dynamic immunological parameter landscape in coronavirus disease 2019 patients with different outcomes. *Frontiers in Immunology* **12**, 697622 (2021).

138 Cotugno, N. *et al.* Virological and immunological features of SARS-CoV-2-infected children who develop neutralizing antibodies. *Cell reports* **34** (2021).

139 Pušnik, J. *et al.* Memory B cells targeting SARS-CoV-2 spike protein and their dependence on CD4+ T cell help. *Cell reports* **35** (2021).

140 Ashokkumar, C. *et al.* Impaired T-cell and antibody immunity after COVID-19 infection in chronically immunosuppressed transplant recipients. *bioRxiv*, 2021.2005. 2003.442371 (2021).

141 Moser, D. *et al.* COVID-19 impairs immune response to Candida albicans. *Frontiers in immunology* **12**, 640644 (2021).

142 Carter, M. J. *et al.* Peripheral immunophenotypes in children with multisystem inflammatory syndrome associated with SARS-CoV-2 infection. *Nature medicine* **26**, 1701-1707 (2020).

143 Wang, F. *et al.* The laboratory tests and host immunity of COVID-19 patients with different severity of illness. *JCI insight* **5** (2020).

144 Koutsakos, M. *et al.* Integrated immune dynamics define correlates of COVID-19 severity and antibody responses. *Cell Reports Medicine* **2** (2021).

145 Zeng, Q. *et al.* Dynamic SARS-CoV-2-specific immunity in critically ill patients with hypertension. *Frontiers in Immunology* **11**, 596684 (2020).

146 Fu, Y. *et al.* Immunological analysis of people in Northeast China after SARS-CoV-2 inactivated vaccine injection. *Vaccines* **9**, 1028 (2021).

147 Du, J. *et al.* Persistent high percentage of HLA-DR+ CD38high CD8+ T cells associated with immune disorder and disease severity of COVID-19. *Frontiers in Immunology* **12**, 735125 (2021).

148 Bobcakova, A. *et al.* Immune profile in patients with COVID-19: lymphocytes exhaustion markers in relationship to clinical outcome. *Frontiers in cellular and infection microbiology* **11**, 646688 (2021).

149 Tomić, S. *et al.* Reduced expression of autophagy markers and expansion of myeloid-derived suppressor cells correlate with poor T cell response in severe COVID-19 patients. *Frontiers in immunology* **12**, 614599 (2021).

150 Gutiérrez-Bautista, J. F. *et al.* Negative clinical evolution in COVID-19 patients is frequently accompanied with an increased proportion of undifferentiated Th cells and a strong underrepresentation of the Th1 subset. *Frontiers in immunology* **11**, 596553 (2020).

151 Rebillard, R.-M. *et al.* Identification of SARS-CoV-2–specific immune alterations in acutely ill patients. *The Journal of clinical investigation* **131** (2021).

152 Lim, J. *et al.* Data-driven analysis of COVID-19 reveals persistent immune abnormalities in convalescent severe individuals. *Frontiers in immunology* **12**, 710217 (2021).

153 San Segundo, D. *et al.* Innate and adaptive immune assessment at admission to predict clinical outcome in COVID-19 patients. *Biomedicines* **9**, 917 (2021).

154 Balzanelli, M. G. *et al.* Immunity profiling of COVID-19 infection, dynamic variations of lymphocyte subsets, a comparative analysis on four different groups. *Microorganisms* **9**, 2036 (2021).

155 Blum, V. F. *et al.* Nitazoxanide superiority to placebo to treat moderate COVID-19–A Pilot prove of concept randomized double-blind clinical trial. *EClinicalMedicine* **37** (2021).

156 Frater, J. *et al.* Safety and immunogenicity of the ChAdOx1 nCoV-19 (AZD1222) vaccine against SARS-CoV-2 in HIV infection: a single-arm substudy of a phase 2/3 clinical trial. *The lancet HIV* **8**, e474-e485 (2021).

157 Alrubayyi, A. *et al.* Characterization of humoral and SARS-CoV-2 specific T cell responses in people living with HIV. *Nature Communications* **12**, 5839 (2021).

158 Lafon, E. *et al.* Potent SARS-CoV-2-specific T cell immunity and low anaphylatoxin levels correlate with mild disease progression in COVID-19 patients. *Frontiers in immunology* **12**, 2171 (2021).

159 Demaret, J. *et al.* Severe SARS-CoV-2 patients develop a higher specific T-cell response. *Clinical & translational immunology* **9**, e1217 (2020).

160 Kaaijk, P. *et al.* Children and adults with mild COVID-19: dynamics of the memory T cell response up to 10 months. *Frontiers in immunology* **13**, 817876 (2022).

161 Hueso, T. *et al.* Convalescent plasma therapy for B-cell–depleted patients with protracted COVID-19. *Blood, The Journal of the American Society of Hematology* **136**, 2290-2295 (2020).

162 Prendecki, M. *et al.* Humoral and T-cell responses to SARS-CoV-2 vaccination in patients receiving immunosuppression. *Annals of the rheumatic diseases* **80**, 1322-1329 (2021).

163 Nilles, E. J. *et al.* Epidemiological and immunological features of obesity and SARS-CoV-2. *Viruses* **13**, 2235 (2021).

164 Rossignol, J. *et al.* Effective Anti–SARS-CoV-2 Immune Response in Patients With Clonal Mast Cell Disorders. *The Journal of Allergy and Clinical Immunology: In Practice* **10**, 1356-1364. e1352 (2022).

165 Bruminhent, J. *et al.* SARS-CoV-2-specific humoral and cell-mediated immune responses after immunization with inactivated COVID-19 vaccine in kidney transplant recipients (CVIM 1 study). *American Journal of Transplantation* **22**, 813-822 (2022).

166 Simon, D. *et al.* Humoral and cellular immune responses to SARS–CoV-2 infection and vaccination in autoimmune disease patients with B cell depletion. *Arthritis & Rheumatology* **74**, 33-37 (2022).

167 Marty, P. K. *et al.* Antigen specific humoral and cellular immunity following SARS-CoV-2 vaccination in ANCA-associated vasculitis patients receiving B-cell depleting therapy. *Frontiers in immunology* **13**, 834981 (2022).

168 Fernández-Ruiz, M. *et al.* Discordance between SARS-CoV-2–specific cell-mediated and antibody responses elicited by mRNA-1273 vaccine in kidney and liver transplant recipients. *Transplantation direct* **7** (2021).

169 Malard, F. *et al.* Weak immunogenicity of SARS-CoV-2 vaccine in patients with hematologic malignancies. *Blood cancer journal* **11**, 142 (2021).

170 Candon, S. *et al.* T cell and antibody responses to SARS-CoV-2: experience from a French transplantation and hemodialysis center during the COVID-19 pandemic. *American Journal of Transplantation* **21**, 854-863 (2021).

171 Lasagna, A. *et al.* A snapshot of the immunogenicity, efficacy and safety of a full course of BNT162b2 anti-SARS-CoV-2 vaccine in cancer patients treated with PD-1/PD-L1 inhibitors: a longitudinal cohort study. *ESMO open* **6**, 100272 (2021).

172 Cassaniti, I. *et al.* Immune response to BNT162b2 in solid organ transplant recipients: negative impact of mycophenolate and high responsiveness of SARS-CoV-2 recovered subjects against delta variant. *Microorganisms* **9**, 2622 (2021).

173 Cucchiari, D. *et al.* Cellular and humoral response after MRNA-1273 SARS-CoV-2 vaccine in kidney transplant recipients. *American Journal of Transplantation* **21**, 2727-2739 (2021).

174 Pompsch, M., Fisenkci, N., Horn, P. A., Kraemer, M. & Lindemann, M. Evidence of extensive cellular immune response after SARS-CoV-2 vaccination in ocrelizumab-treated patients with multiple sclerosis. *Neurological Research and Practice* **3**, 1-6 (2021).

175 Gao, Y. *et al.* Immunodeficiency syndromes differentially impact the functional profile of SARS-CoV-2-specific T cells elicited by mRNA vaccination. *Immunity* **55**, 1732-1746. e1735 (2022).
